# Chiral Fibers Formation Upon Assembly of Tetraphenylalanine Peptide Conjugated to a PNA Dimer

**DOI:** 10.1002/chem.202200693

**Published:** 2022-05-23

**Authors:** Andrea Mosseri, Maria Sancho‐Albero, Marilisa Leone, Donatella Nava, Francesco Secundo, Daniela Maggioni, Luisa De Cola, Alessandra Romanelli

**Affiliations:** ^1^ Dipartimento di Scienze Farmaceutiche Università degli Studi di Milano via Venezian 21 20133 Milano Italy; ^2^ Department of Molecular Biochemistry and Pharmacology Istituto di Ricerche Farmacologiche Mario Negri IRCCS 20156 Milano Italy; ^3^ Istituto di Biostrutture e Bioimmagini – CNR via Mezzocannone 16 80134 Naples Italy; ^4^ Istituto di Scienze e Tecnologie Chimiche “Giulio Natta”, CNR via Mario Bianco 9 Milan 20131 Italy; ^5^ Dipartimento di Chimica Università degli Studi di Milano Via Golgi 19 20133 Milano Italy

**Keywords:** assembly, fiber, helix, peptide, peptide nucleic acid

## Abstract

Self‐assembly of biomolecules such as peptides, nucleic acids or their analogues affords supramolecular objects, exhibiting structures and physical properties dependent on the amino‐acid or nucleobase composition. Conjugation of the peptide diphenylalanine (FF) to peptide nucleic acids triggers formation of self‐assembled structures, mainly stabilized by interactions between FF. In this work we report formation of homogeneous chiral fibers upon self‐assembly of the hybrid composed of the tetraphenylalanine peptide (4F) conjugated to the PNA dimer adenine‐thymine (at). In this case nucleobases seem to play a key role in determining the morphology and chirality of the fibers. When the PNA “at” is replaced by guanine‐cytosine dimer “gc”, disordered structures are observed. Spectroscopic characterization of the self‐assembled hybrids, along with AFM and SEM studies is reported. Finally, a structural model consistent with the experimental evidence has also been obtained, showing how the building blocks of 4Fat arrange to give helical fibers.

## Graphical abstract

**Tetraphenylalanine peptide (4F)** conjugated to the PNA dimer “at” self‐assembles to produce helical fibers. Interactions between both components of the conjugate, either the peptide and the nucleobases, trigger self‐assembly. Antiparallel beta sheets between the peptides are zipped by hydrogen bonds between complementary nucleobases. Spectroscopic measurements, microscopy data along with a computational model support this hypothesis. On the contrary, when “at” is replaced by “gc” PNA, odd shaped spheroid are obtained.

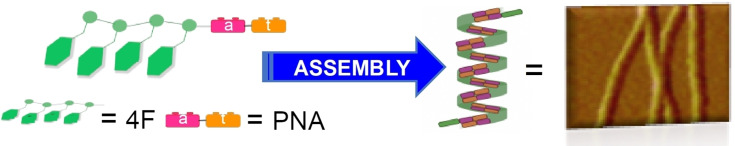

## Introduction

The obtainment of supramolecular structures upon self‐assembly of natural building blocks, such as small peptides, nucleic acids or hybrid systems has attracted intense research interest during last years.^[1]^ These assemblies, in fact, are easy to produce and biocompatible and for these reasons they are particularly suited for biomedical applications. As an example, the hydrogel obtained combining the dipeptide Fmoc‐FF and polyLysine was reported to deliver in vivo, in a controlled fashion at the tumor site the photosensitive drug Chlorin e6. In addition, self‐assembled peptides have been proposed as scaffold for tissue engineering.^[2]^ Chemical composition determines the ability of such building blocks to self‐assemble in aqueous or in organic solvents in a defined secondary structure and in a specific supramolecular arrangement that ultimately determines the morphology of the assembled molecules. In case of peptides, computational studies, supported by experimental evidence, helped to identify general “guidelines” to produce self‐assembled systems. It is known, for example, that hierarchical organization of peptides depends on the amino‐acid sequence (with aromatic amino‐acids being the most prone to aggregation) and on the nature of the N‐capping moiety, that may provide the self‐assembled structure with new functions such as ability to respond to an external stimulus.^[3]^ In case of oligonucleotides, self‐assembly is dictated by Watson‐Crick or Hoogsteen hydrogen bonds between complementary bases, resulting in a variety of structures ranging from duplex, to triplex, quadruplexes, i‐motif or others.^[4]^ However, in case of hybrid systems composed of molecules of different nature, it is very difficult to anticipate their propensity to self‐assemble and also their structure/morphology as it depends on the balance between different intermolecular forces.

In the last few years, the self‐assembly of Peptide Nucleic Acids (PNAs) has also been investigated, because PNAs enclose features of nucleic acids, such as the ability to interact with complementary bases by Watson Crick hydrogen bonds, and features of peptides, such as chemical stability due to the pseudo‐peptide backbone.^[5]^ Compared to nucleic acids, they show a higher propensity to aggregate due to lack of charges on the backbone. First studies reported by Gazit et al. showed that only PNA dimers containing guanine form stable aggregates and that the self‐assembly of monomers was observed only when they were conjugated to aromatic moieties.^[6]^ In another study, Berger et al. observed the formation of photonic crystals from Fmoc(Bhoc) guanine.^[7]^ In a recent report Fmoc guanine conjugated to an alkyl chain resulted in assemblies endowed with an incredible stiffness.^[8]^ Finally, we and others observed aggregation of all PNA monomers and homodimers conjugated to the dipeptide diphenylalanine (FF).^[9]^ In PNA homodimers conjugated to FF, the peptide moiety drives the aggregation while the nucleobases determine the fluorescence properties of the assemblies. The morphology of these self‐assembled systems is highly variable: in water PNA monomers conjugated to FF produce heterogeneous samples, rich in spheroids. On the contrary, PNA homodimers conjugated to the peptide FF form fibers in which peptides are arranged in a beta sheet structure. The PNA heterodimer “gc” conjugated to FF produces mainly spheroids; indeed, samples are not homogeneous, as they contain aggregates of different shape and dimension.^[10]^ Consistent with this observation is the lack of ordered structure in other FF conjugates, containing polyphenylalanine conjugated to the metal chelator DOTA. It was reported that the replacement of FF by 4F resulted in ordered aggregates, in which tetraphenylalanines were arranged in antiparallel beta sheets.^[11]^ Studies on self‐assembly of 4F peptides, either uncapped and capped at the C and/or at the N terminal end in various conditions demonstrated that this peptide is able to form a variety of defined structures.^[12]^ In addition, in conjugates with different polymers such as polyethylene glycol and polylactide, 4F plays a key role in determining the morphology of the aggregate.^[13]^

With the aim to produce supramolecular systems, showing homogeneous structure and dimension, in this work we have explored new PNA‐peptide conjugates, FFFFat (4Fat) and FFFFgc (4Fgc), containing the peptide tetraphenylalanine (4F) conjugated to self‐complementary PNA dimers, i. e., “at” and “gc”.

Absorption, emission spectroscopy and Circular Dichroism (CD), together with microscopy experiments (AFM and SEM) were employed to investigate respectively their optical properties, molecular arrangement, and the morphology of the supramolecular structures. Indeed, emission was observed upon aggregation of nucleobases while the CD evidenced a marked tendency to form chiral fibers by 4Fat molecules, while spheroids mixed with other structures of different shapes and dimensions are obtained with 4Fgc. In these constructs the presence of a tetraphenylalanine peptide favor the formation of ordered aggregates thanks to the extended network of hydrogen bonds and aromatic interactions between peptide chains, as suggested by fluorescence and NMR. Finally, the presence of complementary bases can in principle contribute to the stability of the assembled systems, thanks to Watson Crick and stacking interactions between nucleobases. A computational model in agreement with experimental results suggests formation of ribbon‐like helical fibers for 4Fat, stabilized by both Watson Crick hydrogen bonds between nucleobases and π‐π interactions between phenylalanines.

## Results and Discussion

### Design, Synthesis and assembly of the conjugates

**Figure 1:**
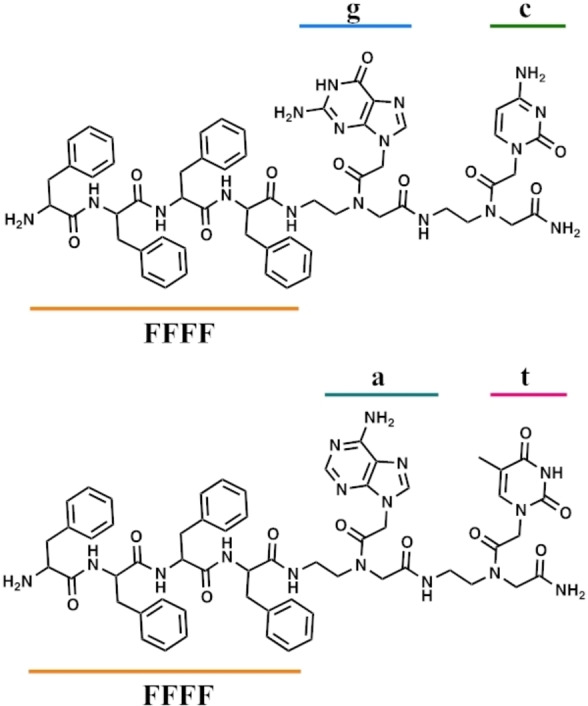
Molecular structures of 4Fgc (top) and 4Fat (bottom).

Chemical structures of the PNA‐peptide conjugates bearing PNA at the C‐terminal end of the molecule are illustrated in Figure 1.

**Figure 2:**
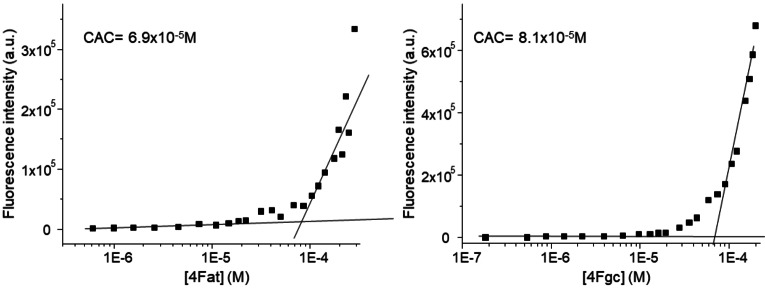
CAC determination: plots of the fluorescence intensity emission of the ANS fluorophore at *λ*=490 nm versus concentration of the 4Fat (left) and 4Fgc (right) conjugates.

The PNA sequences contain complementary bases, “at” and “gc”, that are expected to promote stabilization of aggregates by formation of Watson Crick hydrogen bonds. In all cases peptides are located at the C‐terminal end of the molecules, as previous experiments on FF conjugated to the PNA dimer “gc” at the N or C terminal end showed that aggregation is favored and more ordered structures are obtained when PNA are at the C‐terminus.^[10]^ All molecules were synthesized by solid‐phase synthesis using standard protocols.^[14]^ The synthetic details and the full chemical characterization are reported in the Supporting Information (Figure S1). In water solution the presence of aggregates was investigated by dynamic light scattering (DLS). The DLS experiments were carried out by subsequent dilutions starting from stock solutions of both 4Fgc and 4Fat PNA‐peptide conjugates in a concentration range 6.5×10^−3^–1.8×10^−4^ M. For both compounds, the presence of nanoaggregates was revealed. Nevertheless, while for 4Fat the dilution did not affect the observed monomodal size distribution, showing at all the concentrations only one peak centred at ca. 100 nm (Figure S2A), in the case of 4Fgc the size distribution was bimodal showing two peaks at ca. 75–140 nm and 250–625 nm in dependence to the concentration (Figure S2B). In addition, for 4Fgc the results were poorly reproducible suggesting different aggregation modes and equilibria present in solution. To estimate the Critical Aggregation Concentration (CAC) fluorescence spectroscopy was employed using a probe sensitive to the polarity of the environment. In particular we have selected the dye 1‐anilino‐8‐naphtalene sulfonate (ANS), which possesses a green emission and low quantum yield in polar environments, while a blueshift in the emission maximum along with an increase in its fluorescence in a hydrophobic environment is observed.^[15]^ ANS is commonly employed to detect folding/unfolding processes in proteins, as a marker of amyloids fibrils and recently as a tool to prove aggregation in water of peptides and PNA‐peptide conjugates.^[16]^ Titration of PNA‐4F conjugates in ANS solutions was monitored by fluorescence, recording the increase in the fluorescence intensity of ANS at 490 nm. The changes in fluorescence intensity at increasing 4F‐PNA concentration are reported in the plots in Figure 2. CAC values calculated at the cross of the two discontinuous lines, using Job's method are similar for the two conjugates and their values estimated around 0.1 mM.

These values are very similar to those reported for “gc” conjugated to the peptide FF indicating that the increase of the peptide chain length does not hamper aggregation.

### Secondary structure studies

**Figure 3:**
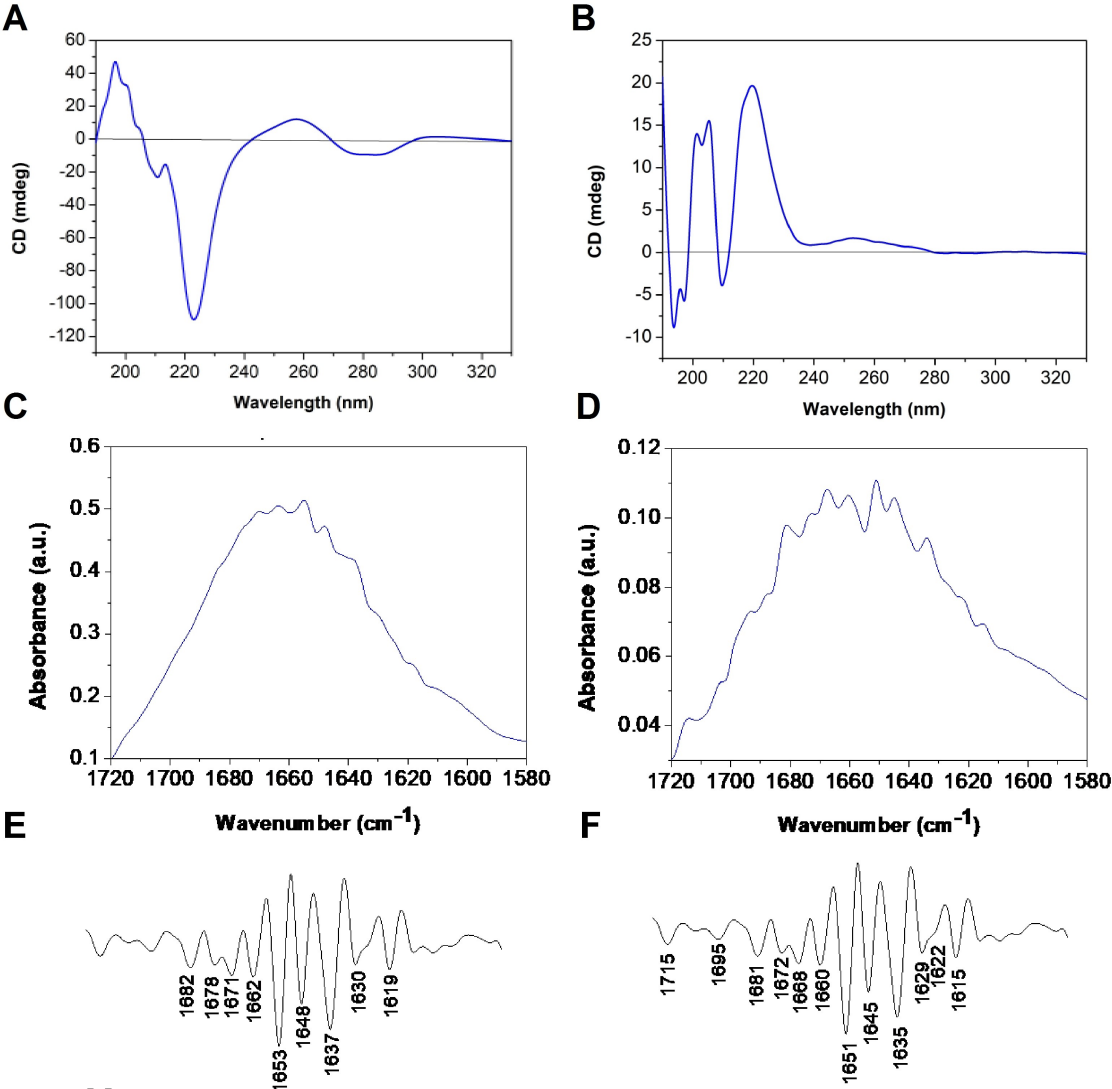
Secondary structure studies: CD spectra in water at (1 mg mL^−1^) of A) 4Fat and B) 4Fgc. FTIR spectra in the amide I region of C) 4Fat and D) 4Fgc. E and F): second derivatives of (C and D).

The secondary structure of the self‐assembled systems was investigated by spectroscopic techniques in water (Figure 3). The pH of all solutions was neutral. Circular Dichroism (CD) spectra were recorded for the two conjugates at different concentrations, namely 1 and at 10 mg mL^−1^. At the lower concentration, the 4Fgc spectrum shows two maxima around 205 and 220 nm attributed to π‐π interactions between phenylalanines; signals at higher wavelengths are significantly weaker (Figure 3B). In case of 4Fat, a minimum at 220 nm is observed suggesting the formation of a beta sheet structure (Figure 3A). Interestingly, spectrum of 4Fat shows signals around 260 and 280 nm; these signals can be attributed to the π‐π* transition of nucleotides and indicate a key role of nucleotides in the stabilization of the structure, as also observed in other peptide‐nucleobases conjugates.^[17]^ In addition, the sign of the bands, positive around 260 nm and negative around 290 nm recalls the Z DNA CD spectrum, that implies formation of a left‐handed helix.^[18]^ At the highest concentration, the spectrum of 4Fat is very similar to that observed at 1 mg mL^−1^ concentration (Supporting Information Figure S3A). When samples for CD were prepared upon dilution with water of concentrated 4Fat and 4Fgc solutions (100 mg/mL) in hexafluoro‐isopropanol (HFIP), 4Fat spectrum shows two intense positive bands around 205 (the most intense) and 220 nm, that suggest aromatic interactions between phenylalanines and weak bands in the region of nucleotides. (Supporting Information Figure S3B). Similar signals were reported for 4F‐PEO and 4F‐PEG conjugates, respectively in THF/water and water solution.[^13b^, ^19^] The 4Fgc spectrum (Supporting Information Figure S3B) shows two positive bands around 198 nm and 220 nm of comparable intensity. In this case we speculate that the lower intensity and the shift of the band at lower wavelength is due to a combination of a negative band at 200 nm typical of random structures and the positive band at 205 nm. In both cases the structure of the assembled systems is different as compared to that observed in water; consistent with data reported in the literature, the supramolecular organization is strongly affected by pathways followed during their formation.^[20]^ In case of beta sheet forming peptides, increase in the percentage of HFIP is related to a shift toward higher random coil percentage. In addition, Fourier Transform Infra‐Red (FTIR) spectra were recorded. Analysis of the amide I region provides useful information on the secondary structure of peptides (Supporting Information Figure S4 and Figure 3). The amide I band in both samples (4Fgc and 4Fat) is not clearly resolved. Thus, in order to enhance resolution and identify the main component bands, we analyzed the vibrational spectrum, transformed the transmittance into absorbance, and calculated the second derivative of each spectrum (Figure 3C and D).^[21]^ Deconvolution of the amide I band reveals multiple signals (Figure 3E and F); bands around 1619 cm^−1^ and 1635 cm^−1^ which are typical of beta sheet structures suggest for our conjugates the presence of intermolecular H‐bond between the amide of the main peptide chain, forming beta sheet structures. Peaks around 1650 cm^−1^ may derive from “disordered” peptides and also from nucleobases. To further prove assembly of our conjugates, we also recorded UV spectra of our samples in the presence of Congo Red. This dye is reported to interact with amyloid structures, showing an increase in the absorption maximum wavelength upon interaction with fibers.^[22]^ In experiments performed incubating Congo Red with 4Fat and 4Fgc the appearance of a maximum at 550 nm is observed, while the maximum of Congo Red alone appears around 500 nm. These results are consistent with the hypothesis of the formation of amyloid‐like aggregates in both cases. (see Supporting Information Figure S5). Also, spectra recorded 3 h after incubation of 4F‐PNA with Congo Red are superimposable to those recorded after 20 min, suggesting that the aggregation process occurs within 20 min (data not shown).

Altogether, CD, UV and FTIR data support the hypothesis that the assembly of the herein developed molecules is related to the interactions between the peptide moiety; consistently with data reported in the literature on other 4F conjugates.[^13b^, ^23^] In 4Fat, the combination of hydrogen bonds between peptide chains and complementary nucleobases contribute to determine the structure of the aggregates: we speculate that nucleobases belonging to different building blocks interact by Watson Crick hydrogen bonds leading to the formation of a helical structure. In 4Fgc, interstrand π‐π interactions and likely to a minor extent hydrogen bonds between amides on peptide chains determine the final structure of the aggregate.

### Fluorescence studies

**Figure 4:**
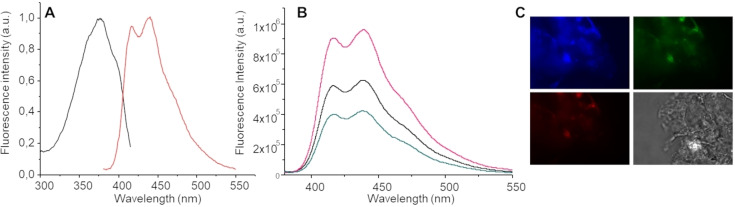
Fluorescence spectra: A) excitation (black) and emission (red) spectra of 4Fat at 1 mg mL^−1^ in water, *λ*_em_=430 nm, *λ*_ex_=360 nm; B) emission spectra at 1.5 (magenta), 1(black) and 0.5 (blue) mg/mL of 4Fat; C) solid‐state emission of 4Fat upon excitation at 330–385 nm (top left), 488 nm (top right), 546 nm (bottom left), bright field (bottom right)

The emission of peptide‐PNA conjugates was analyzed in water solution and in the solid state at different excitation wavelengths. Both conjugates exhibit fluorescence, as shown in Figure 4 and in Supporting Figure S6. Upon excitation at 257 nm we observe fluorescence emissions at 310 nm. The emission is more intense for 4Fgc than for 4Fat and such an emission is attributed to a fluorescence from the aggregated molecules (Supporting Information Figure S6).^[24]^ When samples are excited at 360 nm, a maximum in the emission appears respectively at 410 nm for 4Fgc and at 418 nm for 4Fat (Figures 4 and S6). These values are very similar to those observed in the literature for aggregated nucleobases.^[9b]^ The fact that we find an intense band in the excitation spectra but such a band is not visible in the absorption spectra in our concentrations suggest that the emission is due to species which are generated in the excited state, excimers.

In addition, a second peak is observed around 440 nm, indicating the presence of a network of hydrogen bonds as observed in amyloid fibrils and supports the hypothesis of a beta‐sheet like structure that stabilizes the aggregates. Plots of fluorescence intensity vs. concentration allowed us to determine the CAC values for our compounds, further confirming data calculated by the ANS method (Supporting Information Figure S7).

Upon deposition and drying on a glass slide, samples exhibit fluorescence (Figure 4).

### Morphology studies

**Figure 5:**
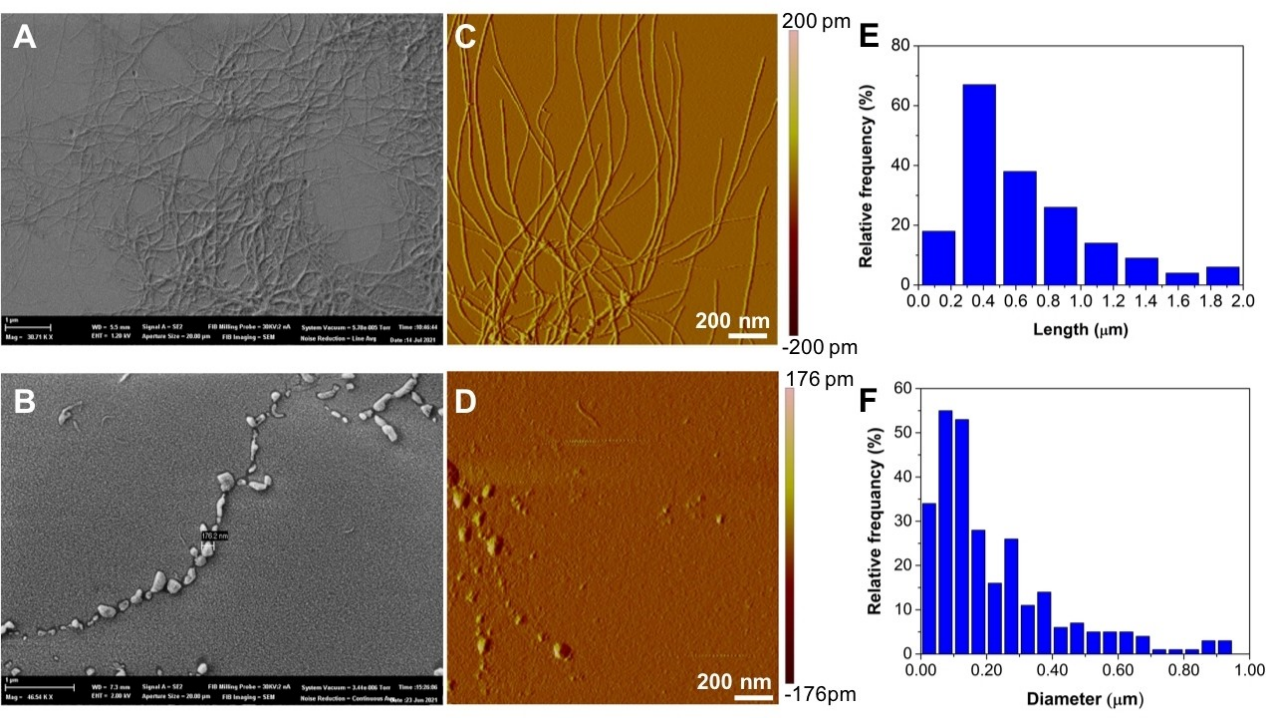
SEM analyses of A) 4Fat and B) 4Fgc in water. Left Tapping mode AFM images of C) the 4Fat and D) 4Fgc samples (amplitude data). E and F) analysis of the distribution of 4Fat fibers length and 4Fgc diameter, respectively.

Morphology of our self‐assembled systems was investigated by both Atomic Force Microscopy (AFM) and Scanning Electron Microscopy (SEM) (Figures 5 and S8). AFM studies were performed to evaluate and clarify the morphology of the 4Fat and 4Fgc assembled in water. Figure 5 shows AFM images of 4Fat and 4Fgc aggregates obtained in identical experimental conditions upon deposition on a muscovite mica disk of 10 mg mL^−1^ solutions in water. It can be clearly seen that the morphology of the two supramolecular structures is completely different: 4Fat formed visible fibers, whereas the 4Fgc system did not exhibit homogeneous fibers and only small heterogeneous aggregates could be visualized (Figure 5). Morphology of 4Fgc is similar to that observed for FFgc.^[10]^ The 4Fat fibers were arranged in an extended way having a broad distribution of width and length (Figures 5A, C, E and S8): most fibers are 400 nm long and show a width in the range 12–15 nm. In order to evaluate the chirality of 4Fat fibers, acquisitions with a higher magnification were recorded. Figure S8 includes images relative to 10 mg mL^−1^ of 4Fat in which helices can be clearly observed. The formation of helical fibers is in agreement with the CD spectra, that suggest formation of left‐handed helical structures when 4Fat is dissolved in water (1 and 10 mg mL^−1^).

SEM images of the two different samples investigated (Figure 5A and B) confirmed AFM results, indicating that although 4Fat sample clearly formed fibers, 4Fgc were not able to assemble giving rise to chains and only big aggregates without a defined structure.

### Molecular modeling studies

Secondary structure studies, as well as microscopy studies revealed the formation of fibers in case of 4Fat, while disordered/irregular structures were obtained in case of 4Fgc. To get further insights into structural features characterizing these aggregates, speculative 3D structural models were built through computational molecular modeling techniques.

**Figure 6:**
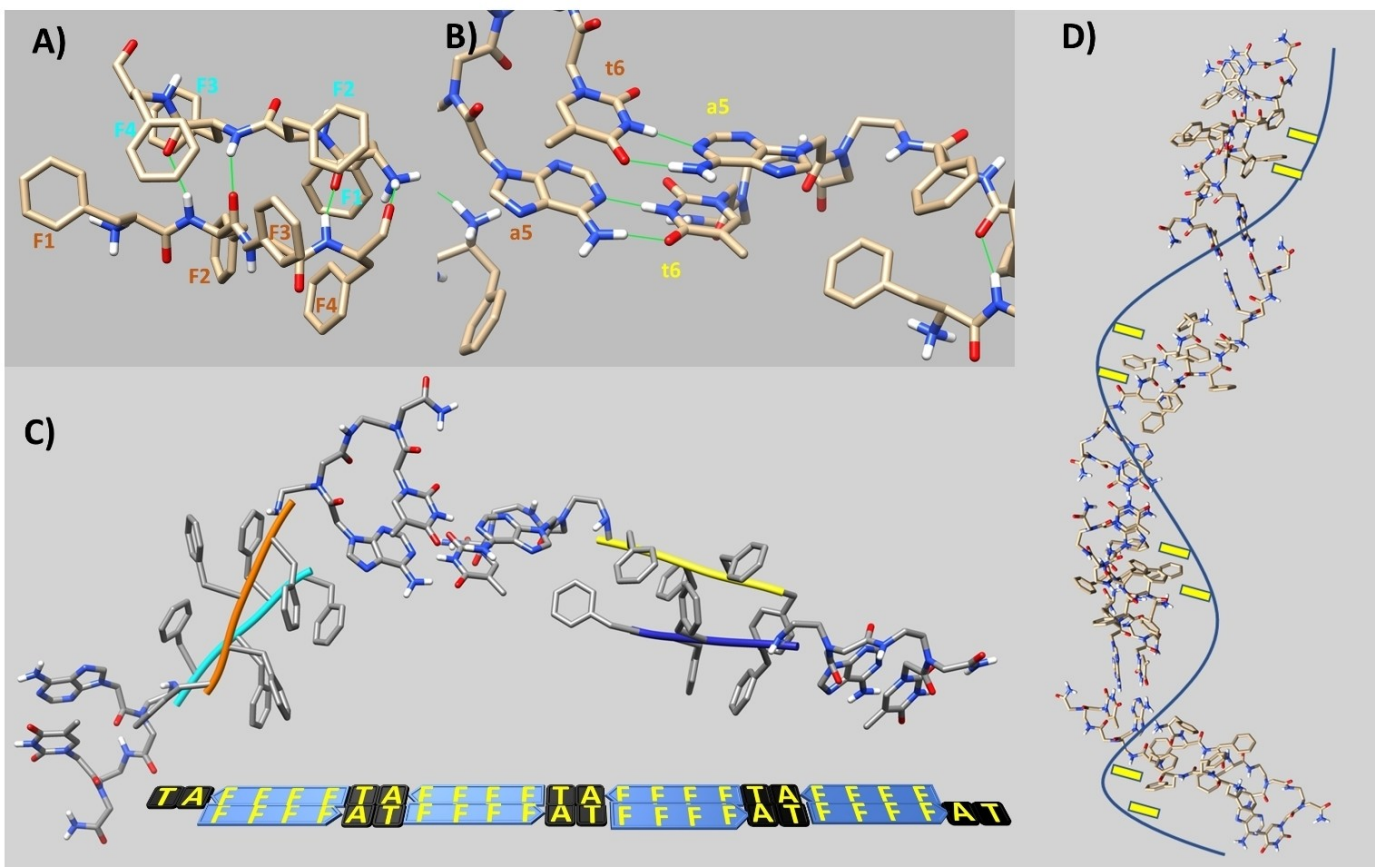
3D structural model of 4Fat assembly. A) Dimeric 4F structural arrangement. Intermolecular H‐bonds in between backbone atoms are indicated by green lines. Phenylalanine side chains are labeled by the one residue amino acid code and sequence numbers, different colors are used for F belonging to different monomeric units. B) AT Watson and Crick canonical base pairing in adjacent 4Fat units. C) Assembly of a 4Fat tetramer. Two antiparallel β‐sheets made up by two dimeric 4F entities are connected by the AT … TA base pairing. Additional 4Fat monomers can be assembled on both edges of the tetramer by exploiting Watson and Crick hydrogen bonding in between PNA bases. D) 4Fat bilayer made up of eight monomeric units. Fibers could arise by twisting bilayers together. The line indicates peptide backbone while the rectangles point out potential docking sites made up by Phe aromatic rings.

For 4Fat we first focused on the interaction between the canonical 4F peptide units. CD spectra point out β‐secondary structure content, thus the software Chimera was employed to generate the structure of a single 4F peptide layer in a β‐conformation.^[25]^ The β‐peptide was subsequently used as input receptor and ligand in Haddock to predict a model of a dimeric 4F entity through docking techniques. 200 structures were calculated by Haddock and 147 of them could be grouped into 12 clusters, the most relevant structure of the best cluster was next considered as representative model (Figure 6).^[26]^ The dimeric 4F structural organization is made up by an antiparallel β‐sheet, this assessment is energetically favored as it localizes the positively charged N‐terminals of single monomers far away from each other thus avoiding steric repulsions; backbone H‐bonds in between the two 4F strands stabilize the structure along with intermolecular π‐π interactions in between phenylalanines side chains facing each other on the two strands (Figure 6A). Starting from the dimeric 4F assembly the at/ta PNA units were added at the C‐termini of each 4F tetrapeptide chain after extrapolating the atomic coordinates from an eight‐mer PNA duplex, that was available in the PDB databank (pdb entry code 2 K4G, Figure 6B).^[27]^ Interestingly, our CD data indicate a left‐handed helical organization in 4Fat similarly to the one observed by CD for the eight‐mer PNA duplex (pdb entry code 2K4G) that we took as reference to build our model.^[28]^ A long bilayer of 4Fat units could be assembled through formation of antiparallel β‐sheets composed by the 4F core in different monomers, characterized by extensive backbone H‐bonds and π‐π interactions, flanked on each side by at/ta Watson and Crick base pairing involving PNA dimers (Figure 6C and D). Several bilayers might spatially assemble into larger aggregates by exploiting the β‐sheet units and further π‐π interactions in between aromatic rings that are available on both sides of the bilayers. The peptide backbones in the bilayers by alternating the β‐conformation of 4F and the left‐handed helical organization of the at/ta PNA units might interact in between each other by twisting together in some sort of superhelical arrangement. The resulting structural organization could give rise to the ribbon‐like helical fibers previously observed by AFM (Figure 5). Although rather speculative, the described computational model is in good agreement with the experimental data and a similar scenario has indeed already been reported for nanostructures formed by dipeptides decorated with naphthalene diimide.^[29]^

In the case of 4Fgc due to lack of information deriving from AFM and CD data, NMR studies were also carried out to investigate possible interactions between conjugates and get further structural insights.

1D [^1^H] and 2D [^1^H, ^1^H] spectra were recorded for 4Fgc at a concentration higher than the CAC; however, it was not possible to explore high concentrations due to relevant precipitation phenomena. High molecular weight oligomers and protofibrils species are NMR invisible while disaggregated and/or small oligomers can be observed by solution NMR.^[30]^ Analysis of the NMR 1D [^1^H] and 2D [^1^H, ^1^H] TOCSY spectra in the region close to 5 ppm (Supporting Information Figure S9) soon shows the appearance of 4 doublets representing signals from the H5 aromatic proton of cytosine (Supporting Scheme S1).^[31]^ In PNA dimeric systems in fact restricted rotation around the tertiary amide gives rise to four rotamers (See Supporting Information Scheme S2) provided with the side chain carbonyl groups of the PNA skeleton oriented either towards the N‐terminal or the C‐terminal sides. NMR data indicate that the gc PNA unit behaves in the 4Fgc compound as isolated PNA segment with one of the rotamers more abundant than the others (Figure S9).^[32]^ However, the NOESY spectrum of 4Fgc reveals several negative contacts and consequently a certain rigidity of the whole system due to aggregation phenomena (Figure S10). The NMR spectra also contain at least double signals for the phenylalanines spin systems further highlighting a strong conformational variability. Comparison of TOCSY and NOESY allowed to achieve almost complete resonance assignments for the most abundant rotamer in solution (Table S1 and Figure S11). For this rotamer side chain CO are likely oriented towards the C‐terminal side as pointed out by the presence of strong NOE contacts between the H81‐H82 side chain protons and the backbone H31‐H32 protons in both g and c and weaker contacts between H81‐H82 and H51‐H52 protons (Figure S11 and Scheme 2). Indeed, a more intense H81‐H82 contact with the aliphatic H5 protons in the c unit slight avoids a perfect orientation of the CO towards the C‐terminal side (Figure S12 and Scheme S2).^[32a]^ However, this distortion can be linked to the higher degree of freedom of the C‐terminal PNA fragment.

It was not possible to unambiguously assign the whole phenylalanine aromatic side chains due to extensive spectral overlaps. Nevertheless, a certain line broadening could also be noticed in the aromatic region of phenylalanines (Figure S10) indicating that they may be more involved into intermolecular contacts at the aggregate interfaces in contrast to the gc termini that remain more flexible and still able to assume multiple rotameric states.

The NOE pattern for the principal rotamer is mainly canonical of an extended or disordered specie. Interestingly weak contacts in between aromatic proton of g5 and those of c6 could be observed in the NOESY spectrum. However, the NMR analyses do not indicate gc canonical base pairing as the imino H_N_ and the amino _N_H2 protons of the g and c bases could not be detected likely due to high solvent exposure. The first backbone H_N_ cannot be observed as well due to high solvent exposure whereas, four different peaks could be detected for the C‐terminal amide group reflecting the diverse gc rotameric states (Figure S9).

The observed NOE contacts were employed to conduct structural calculations and to get insights into the conformational space that is accessible to monomeric 4Fgc units. The NMR solution structure was calculated with the software CYANA by employing 125 distance restraints (79 intra‐, 36 short‐, 10 medium‐range contacts) and 21 angle restraints (Figure S13).^[33]^

These structure calculations highlighted the tendency of the N‐terminal aminoacidic portion to assume a more ordered and partially extended conformation. The RMSD on all atoms from residues 1 to 4 is 0.672 Å while it increases to 2.048 Å if the PNA dimer gc is included. The 20 conformers were subjected to a clusterization procedure to identify subfamilies of conformationally related structures (Table S2) and this analysis further stressed out the conformational variability.[^25^, ^34^] In fact, three main clusters could be retrieved including 10/20 structures with the most abundant one including only 5 conformers (Figure S8 and Table S2) while the remaining 10 structures over the 20 in the NMR ensemble behave as unrelated conformers.

The three representative structures of the most abundant clusters (number 12, 1 and 16) (Table S2) were further employed to get structural insights into possible dimeric units that could constitute 4Fgc aggregates. The best solution in term of predicted binding energy is shown in Supporting Figure S14. This speculative model is characterized by π‐π interactions involving F2 and F4 from one 4Fgc unit and F4 from the other chain assuming a sandwich‐like arrangement; additional π‐π intermolecular contacts occur between F1 and F2 and F3 and F1 (Figure S14). Moreover, the dimeric arrangement is stabilized by three intermolecular H‐bonds, two of which involving backbone H_N_ of F3 and F2 with the _C_O of F1 and another one involving the amino group of g5 and the carbonyl oxygen of g5 ring into distinct monomeric units. Stacking interactions in between aromatic rings, that generate extensive sandwich‐like arrangements, are recurrent in different docking solutions. Thus, computational docking studies, although speculative, appear in line with NMR experimental data and highlight that π‐π contacts are mainly driving self‐assembly of 4Fgc. Such a structure organization could give rise to an aromatic hydrophobic inner core that is masked from the solvent while the gc PNA dimers remain solvent exposed able to assume different rotameric states. Docking studies, again in agreement with NMR data do not let speculate gc canonical base pairing in the aggregates.

## Conclusion

Building blocks that self‐assemble in a unique manner are desirable to achieve highly ordered supramolecular structures. When using hybrid systems composed of different biomolecules, such as amino acids and Peptide Nucleic Acids it is usually very hard, if not impossible, to foresee which forces will determine self‐assembly, although interactions between single components have been widely explored. Formation of “disordered” aggregates, showing multiple shape and dimensions, is very common in hybrid systems. Herein we have demonstrated that it is possible to obtain homogenous fibers upon self‐assembly of a hybrid molecule composed of the peptide tetraphenylalanine conjugated at the C‐terminus with the PNA dimer “at”. 4Fat forms ribbons, as demonstrated by AFM in which peptides are arranged in antiparallel beta‐sheets. According to the speculative structural model obtained by computational studies, nucleobases “at” work as a zipper for building blocks, that arrange to give 400 nm long fibers. On the contrary, 4Fgc aggregates to give irregular elongated objects, with dimensions ranging from tens to hundreds of nanometers. In this case, as observed for 4Fat, experimental results suggest that the peptide might still arrange in a beta sheet fashion, and that extensive intermolecular π‐π contacts between phenylalanine side chains might establish but the PNA dimer gc does not hybridize to another PNA dimer gc on another strand. For this reason, fibers are not formed. Irregular shapes observed for 4Fgc very much recall those observed for molecules containing the gc dimer conjugated to the peptide FF. The reason why “at” triggers fiber formation unlike “gc” is still to be cleared. However, it cannot be excluded that the presence of a methyl group in the thymine could provide additional hydrophobic contacts and interactions with π‐systems in either bases and phenylalanine side chains thus supporting formation of well‐organized fibers.

Indeed, the finding that the combination of 4F peptide and “at” PNAs in a single molecule results in a building block able to assemble in a defined fashion represents a valuable starting point for the design of new functional biomaterials, whose properties can be tuned exploiting specific and tunable interactions of their components.

## Materials and Methods

### Solid phase synthesis

The conjugates were synthesized in a 20 μM scale using solid phase Fmoc chemistry by standard protocols on the Rink amide resin.^[14b]^ After the last coupling, the deprotection of the N‐terminus was performed using 30 % piperidine in DMF (7 min for PNA, 5 min (2 times) for amino acid), the resin was washed with DMF (3×) and DCM (3×) and dried *in vacuo*.

Cleavage from the resin and deprotection of the conjugates was performed by treating the dry resin with a solution containing 90 % TFA/ 10 % TIS (v/v) for 90 min under shaking. The resin was filtrated; the solution was flushed by a stream of nitrogen to remove most of the TFA; the peptide‐PNA was then precipitated by addition of cold diethyl ether. The precipitate was washed three times, the *crude* was dissolved in water and lyophilized. Samples were analyzed and purified by RP‐HPLC using a gradient of CH_3_CN (0.1 % v/v TFA) in H_2_O (0.1 % v/v TFA) from 10 % to 50 % in 20 minutes, preceded by 2 min equilibration. For the purification a Jupiter® 10 μm Proteo 90 Å, LC Column 250×4.6 mm, Ea was used, for the analysis the column Sepachrom Vydamas 5 μ C18 100 Å 150×4.6 mm was employed. Samples were identified by mass spectrometry, using an Applied Biosystems 4700 Proteomics Analyzer instrument.

After RP‐HPLC purification, the products were lyophilized three times. The first one to remove the solvents used in HPLC, the second one using a solution of H_2_O/CH_3_COOH 7 : 3 v/v and the third one using only H_2_O.

4Fgc sequence: FFFFgc

Retention time=16.55 min. Calculated mass (Da) for [M+Na]^+^ 1170.4992; found: 1170.9336;

4Fat sequence: FFFFat

Retention time=17 min. Calculated mass (Da) for [M+H]^+^=1147.5219 and [M+Na]^+^=1169.5038; found: 1147.8032 and 1169.6400.

### UV‐visible absorption

The UV‐vis absorption measurements were conducted on a spectrophotometer Jasco V‐530. UV spectra were acquired in a range of 240–350 nm; the absorbance values at 260 nm were employed to evaluate the samples concentration. Extinction coefficient (*ϵ*) values for the PNA‐peptide conjugates calculated based on the values reported in the literature of each base and amino acid are 19635 for 4Fgc and 24175 for 4Fat M^−1^ cm^−1^. The concentration was calculated applying the Lambert‐Beer law.

### DLS measurement

The Dynamic Light Scattering (DLS) measurements were performed using a Malvern Zetasizer Nano ZS instrument at 25 °C, equipped with a 633 nm solid state He−Ne laser at a scattering angle of 173°. Analyses were conducted in water (viscosity: 0.8872 Cp, refractive index: 1.33). The size measurements were averaged from at least three repeated measurements. Solution for DLS measurements were obtained by dilution in water of the 100 mg mL^−1^ HFIP solution of the samples. Measurements were performed on each sample at a different concentration. The data were plotted using Prism software.

### CAC determination

Solutions in water at increasing concentration of 4Fat and 4Fgc were titrated into 200 μM solution of ANS. Fluorescence emission spectra were recorded on a Fluorolog Jobin Yvon Horiba instrument; excitation wavelength was set at 350 nm. Fluorescence emission at 490 nm was plotted vs. PNA‐peptide conjugate concentrations. The experiment was repeated in duplicate. CAC were calculated as reported in the literature.^[9b]^

### Circular dichroism

The secondary structure of assembled 4Fat and 4Fgc samples was determined by circular dichroism spectroscopy. The circular dichroism spectra were recorded using a Jasco J‐815 spectropolarimeter (Jasco, Easton, MD) at 25 °C from 190 nm to 400 nm (1 nm bandwidth and 0.1 nm resolution). The measurements were executed in a 0.1 mm or a 0.1 cm optical path quartz capillary cuvette at 25 °C. Samples were prepared from dilution (1 : 100) in water of 100 mg mL^−1^ solutions of samples in HFIP. In different experiments samples were dissolved in water and the analyzed at 10 and 1 mg mL^−1^. The temperature in the cuvette was regulated with a Neslab RT‐11 circulating water bath. Each sample was measured in triplicate. CD spectra are measured in units of mdeg as a function of wavelength.

### Congo red

Congo Red (CR) spectroscopic assay was carried out by UV‐Vis measurements on a Shimadzu UV‐3600 spectrophotometer. A stock solution of CR (3,5 mg in 500 μL) was freshly prepared in 10 mM phosphate buffer, at pH 7.4 and filtered through 0.2 μm syringe immediately prior to use. A small aliquot (5 μL) of this solution was diluted with water at 12.5 μM final concentration and the UV‐Vis spectrum was recorded between 400 and 700 nm at room temperature. 60 μL of each PNA‐peptide solution (20 mg mL^−1^) were added to CR solution to obtain a final peptide concentration of 2.5 mg mL^−1^. The sample was left in incubation for 20 min at room temperature, then absorbance spectra were recorded and background subtracted using Congo Red spectrum in phosphate buffer as reference solution.

### FTIR

FTIR spectra were recorded using a Shimadzu IR Affinity‐1 spectrometer. The transmittance spectrum was collected using a spectral resolution of 1 cm^−1^, accumulating 45 scans and the ATR‐FTIR spectra were ATR corrected (penetration depth mode). The FTIR spectra of the samples were collected with 200 scans in the 600–4000 cm^−1^ spectral region.

### Fluorescence

Solutions for fluorescence measurements were obtained dissolving samples in water at a final concentration of 5 mg mL^−1^. The experiments were conducted on a spectrofluorometer Fluorolog Jobin Yvon Horiba using 1 cm path length. The emission spectra were registered exciting at different wavelengths in a range between 300–390 nm; the excitation spectra were acquired at different emission wavelengths from 410 to 436 nm. Excitation and emission spectra were acquired using the same slits parameters, each product needed different slits. The data plotting was performed by OriginLab software.

### AFM

Both 4Fat and 4Fgc samples were dissolved in H_2_O (1 and 10 mg mL^−1^) and 50 μL were spotted onto a freshly cleaved Muscovite mica disk and incubated for 5 min at room temperature. The disk was washed with H_2_O, and it was finally dried under a gently nitrogen stream during 5 min.

Muscovite mica disk containing the samples were placed onto a Multimode AFM with a NanoScope V system (Veeco/Digital Instruments) operating in Tapping Mode using standard antimony(n)‐doped Si probes (T: 3.5–4.5 mm, L: 115.135 mm, W: 30–40 mm, f0: 313–370 kHz, k: 20–80 N/m) (Bruker). Samples were analyzed with the scanning Probe Image Processor (SPIP Version 5.1.6 (released April 13, 2011) data analysis package. SPIP software was also employed to analyze the chirality of the fibers.

### SEM

Coated specimens were observed through SEM using secondary electron detection (Cross‐Beam 1540EsB electron microscope (Supra55, Carl Zeiss)). Acceleration voltage was set to 0.4 to 1.5 kV, working distance to 4 to 6 mm and enlargement up to 20 kx. Multiple SEM images at various magnifications were acquired for both studied samples.

### Optical microscopy

5 μL of 4Fat and 4Fgc (10 mg mL^−1^) were deposited onto a slip before being imaged. Pictures of 4Fat and 4Fgc were acquired with an Olympus Microscope IX71 (Olympus, Shinjuku, Japan) with Cell F (2.6 Build1210, Olympus, Shinjuku, Japan) imaging software

### NMR

NMR solution structure studies were conducted for a 4Fgc sample at a concentration of 4 mg mL^−1^. The sample was prepared by dissolving compound powder in 540 μL of H_2_O: D_2_O (v : v) (90 : 10) or pure D_2_O (Deuterium Oxide, 98 % D, Sigma‐Aldrich, Milan‐Italy). NMR spectra were recorded at 298 K on a Bruker Avance 600 MHz spectrometer.

The following set of experiments was recorded: 1D [^1^H], 2D [^1^H, ^1^H] TOCSY (Total Correlation Spectroscopy), NOESY (Nuclear Overhauser Enhancement Spectroscopy), ROESY (Rotating frame Overhauser Enhancement Spectroscopy) and DQFCOSY (Double Quantum‐Filtered Correlated Spectroscopy).^[35]^ 2D spectra were usually recorded with 8–16 scans, 512 FIDs in t1, 2048 data points in t2. In addition, the TOCSY spectrum was acquired with a mixing time equal to 70 ms, NOESY spectra were recorded with mixing times of 200 and 300 ms and the mixing time for ROESY experiment was set to 250 ms. Excitation Sculpting was employed to achieve water suppression. Chemical shifts were referenced to the residual water peak at 4.7 ppm.^[36]^ Spectra were processed with Topspin 2.1 (Bruker, Italy) and analyzed with the software MestReNova and NEASY contained in CARA (http://www.nmr.ch/).^[37]^

### Solution structure calculation

The NMR solution structure of 4Fgc was calculated with CYANA 2.1.^[33]^ Peaks in the 2D [^1^H, ^1^H] NOESY 300 spectrum were manually integrated and converted into distance constraints in the form of upper distance limits by the CALIBA routine of CYANA.^[33]^ The GRIDSEARCH module of CYANA was next implemented to derive angle constraints. CYANA standard residue library was modified to insert the PNA gc dimer: PNA g and c library entries were generated from pdb files employing CYLIB.^[38]^ Structure calculations initiated from 100 random conformers, next, the 20 conformers with the lowest target functions and thus, in better agreement with distance and angle constraints, were chosen as representative models and included in the NMR ensemble. The 20 final structures were further inspected with the programs MOLMOL and UCSF‐Chimera.[^25^, ^39^] UCSF‐Chimera was implemented to identify clusters of conformationally related subfamilies.^[25]^ The ensemble cluster module of Chimera was run by matching all atoms in residues from Phe1 to c6.^[34]^

### Computational studies

#### 4Fat

The 4F peptide was built in UCSF Chimera version 1.14 assuming an antiparallel β‐strand structural organization (*ϕ* −139°‐*ψ* 135°).^[25]^ This tetrapeptide was used as input in Haddock (Haddock2.4 webserver) to generate atomic coordinates of a dimer.^[26]^ The N‐terminal was considered positively charged while the four Phe residues were set as active during docking calculations. The docking protocol included first a rigid body energy minimization during which 1000 structures were calculated. Next a semi‐flexible simulated annealing of the best 200 solutions was carried out followed by a final refinement in water. In the end a clusterization procedure of the obtained 200 models was conducted (RMSD cut‐off value of 5 Å).^[26]^ The best structure of the best cluster (i. e., number 1 from cluster 2 with Haddock score equal to −69.0601) was chosen as representative model and employed to assemble multiple 4Fat copies. The atomic coordinates for the PNA duplex AT…TA were extracted from the pdb entry 2 K4G and joint to the Haddock 4F model by creating peptide bonds in Chimera with default parameters (C−N length 1.33 Å, *ω* angle equal to 180°) between the backbone NH amide group of adenines and the F4 CO groups.^[27]^ In such a way eight FFFFat monomeric units were combined together. The resulting aggregate was subjected to unrestrained energy minimization with the MMTK (Molecular Modelling Toolkit) minimization module of UCSF Chimera including 1000 steepest descent cycles and 100 conjugate gradients cycles (step size 0.02 Å).^[28]^

#### 4Fgc

Docking studies were performed with AutoDock Vina (version 1.1.2).^[40]^ For docking studies CYANA structures were subjected to an unrestrained energy minimization (100 steps of steepest descent and 10 steps of conjugate gradient) with UCSF‐Chimera.^[25]^ Three separate virtual screening runs were carried out using as receptor entities the energy minimized CYANA structures n.1, 12 and 16 for run 1, 2 and 3, respectively. Phenylalanine side chains in the receptors were considered flexible in each run. CYANA structure n.1, 12 and 16 were employed as ligands for virtual screening as well. The .pdbqt files necessary to run AutoDock Vina were generated starting from the corresponding .pdb files with ADT.^[41]^ The Vina grid was designed large enough to include all four phenylalanine residues. To ensure reproducibility of our solutions, the number of seed was set at the same value in all screening runs.^[42]^ The configuration parameter “exhaustiveness”, that establishes how many times the same run is repeated, was set to 16.^[42]^ The maximum number of output binding poses was set to 1.^[42]^ The solutions with best Vina scores were analyzed with UCSF‐Chimera and MOLMOL.[^25^, ^39^, ^40^]

## Conflict of interest

The authors declare no conflict of interest.

1

## Supporting information

As a service to our authors and readers, this journal provides supporting information supplied by the authors. Such materials are peer reviewed and may be re‐organized for online delivery, but are not copy‐edited or typeset. Technical support issues arising from supporting information (other than missing files) should be addressed to the authors.

Supporting Information

## Data Availability

The data that support the findings of this study are available from the corresponding author upon reasonable request.
