# Supplementary material for: Chiral Fibers Formation Upon Assembly of Tetraphenylalanine Peptide Conjugated to a PNA Dimer
Source: Chemistry. 2022 May 23;28(37):e202200693. doi: 10.1002/chem.202200693 (PMC9325372; doi:10.1002/chem.202200693)
Supplement: Supplementary file 1 — Supporting Information [file CHEM-28-0-s001.pdf]

Chemistry–A European Journal

Supporting Information

Chiral Fibers Formation Upon Assembly of Tetraphenylalanine Peptide Conjugated to a PNA Dimer

Andrea Mosseri, Maria Sancho-Albero, Marilisa Leone, Donatella Nava, Francesco Secundo, Daniela Maggioni, Luisa De Cola, and Alessandra Romanelli*

Author Contributions

A.M. Investigation:Lead
M.S.-A. Investigation:Lead
M.L. Data curation:Equal; Formal analysis:Lead; Investigation:Equal
D.N. Investigation:Supporting
F.S. Investigation:Supporting
D.M. Formal analysis:Supporting; Investigation:Supporting
L.D. Resources:Equal; Supervision:Lead; Writing – review & editing:Equal
A.R. Conceptualization:Lead; Supervision:Lead; Writing – original draft:Lead

Supplementary material

Contents

	page
Figure S1. HPLC and MS of 4Fat and 4Fgc	2
Figure S2. DLS measurements	4
Figure S3: CD spectra	5
Figure S4: FT-IR spectra	6
Figure S5: UV spectra of Congo Red	7
Figure S6: Fluorescence spectra	8
Figure S7: CAC determination by fluorescence measurements	9
Figure S8: AFM analysis of 4Fat	10
Scheme S1: Atom nomenclature	11
Scheme S2: Chemical structures of different rotamers	11
Figure S9: Expansion of the 1D ^{1}H spectrum of 4Fgc	12
Figure S10: 2D [^{1}H, ^{1}H] NOESY300 spectrum of 4Fgc	13
Figure S11: Comparison of backbone HN/high field correlation regions in TOCSY and NOESY spectra of 4Fgc	14
Figure S12: Region of the NOESY 300 spectrum	15
Figure S13A: Ensembles of different conformational families	16
Figure S13B: CYANA structures	16
Figure S14: Representative model of the 4Fgc dimer	16
Table S1: ^{1}H Chemical shifts (ppm) of 4Fgc main conformer	17
Table S2: Cluster analysis of 4Fgc structures.	18
References	18

Figure S1. HPLC and MS of 4Fat and 4Fgc

a. HPLC profile and mass spectrum of pure 4Fat

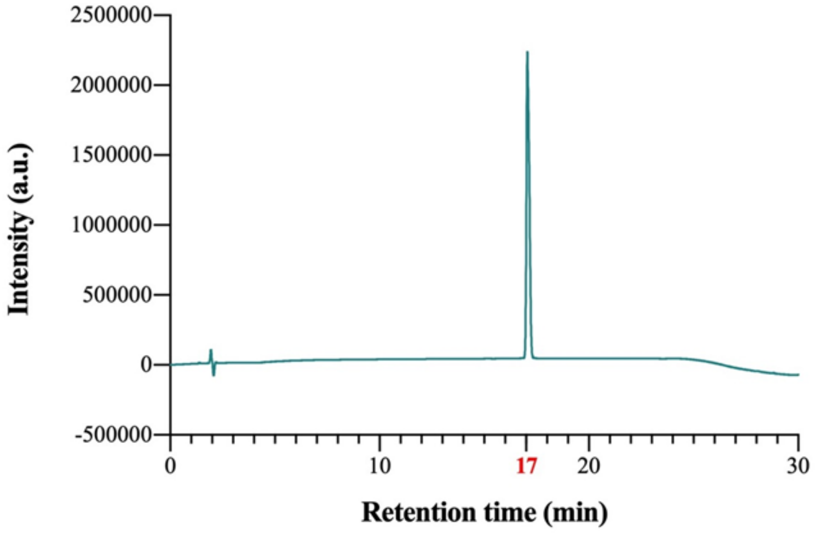

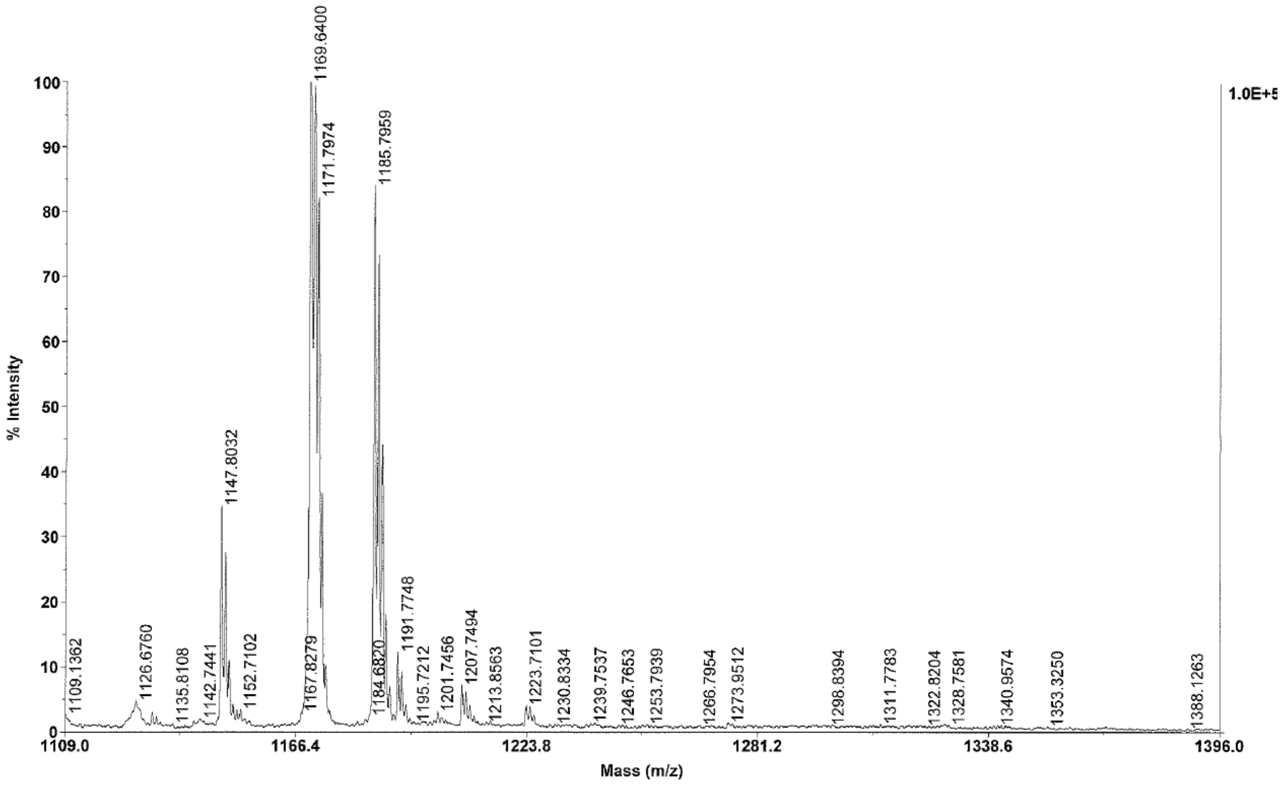

b: HPLC profile and mass spectrum of pure 4Fgc

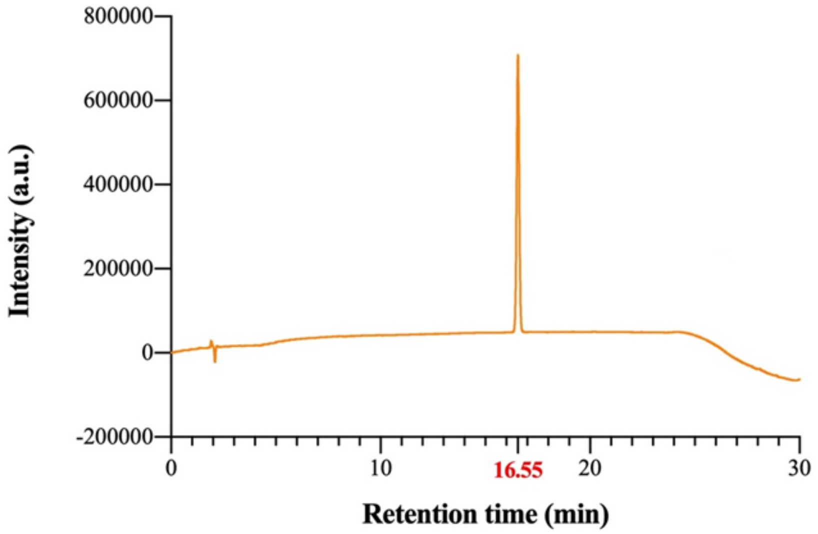

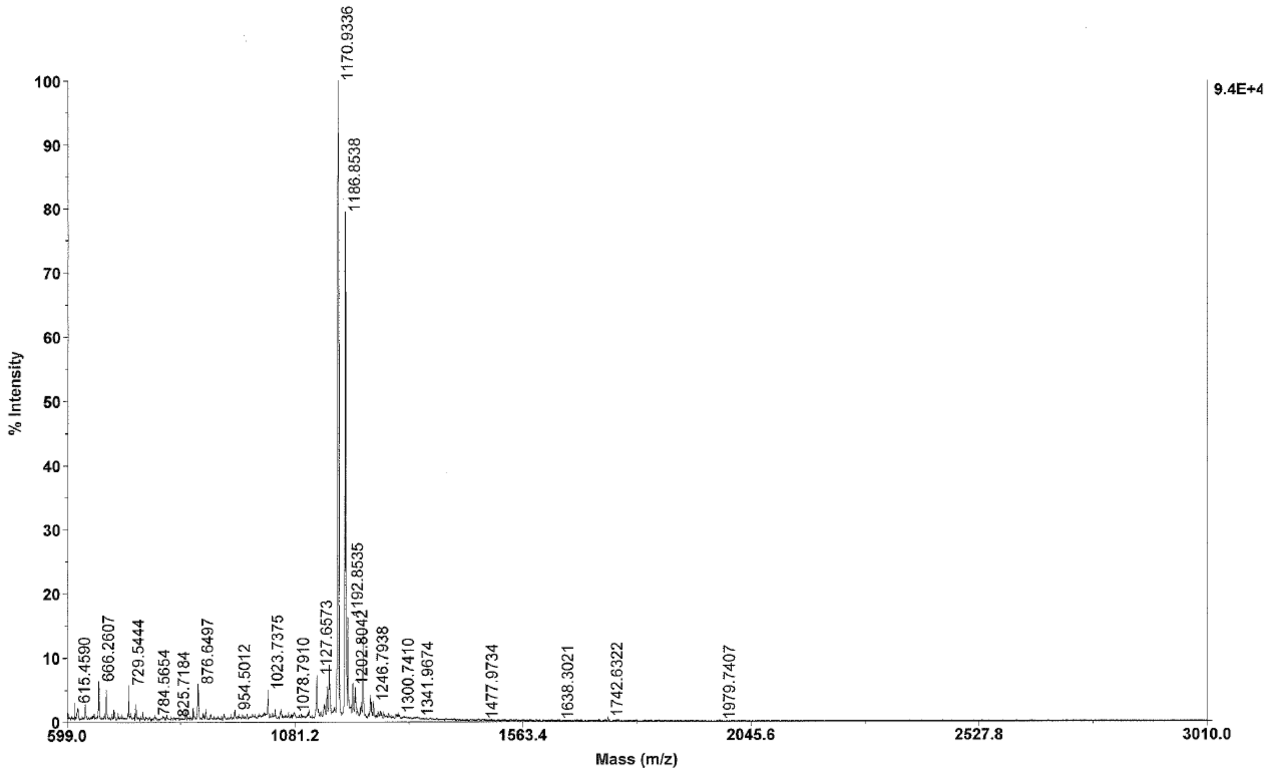

Figure S2. DLS measurements (by intensities) of (**A**) 4Fat and (**B**) 4Fgc at different concentrations at 25 °C.

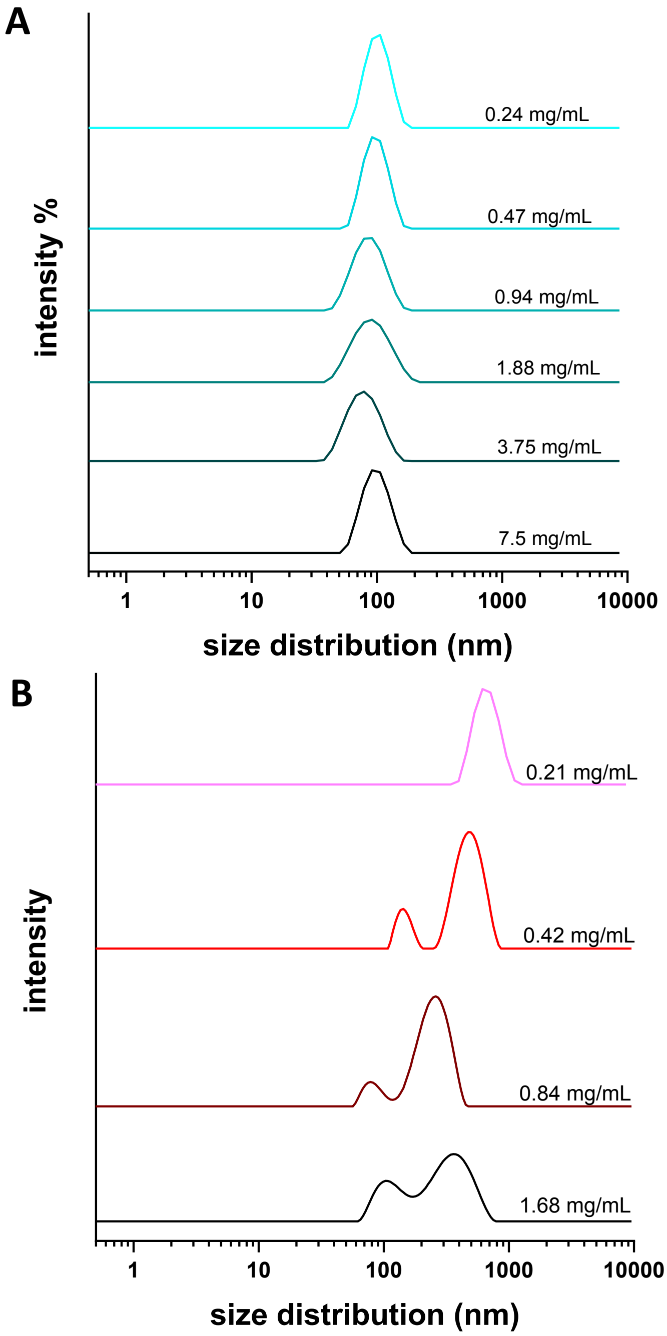

Figure S3: A) CD spectrum of 4Fat (10 mg/mL) in water; B) CD spectra of 4Fat (red) and 4Fgc (blue) in water/HFIP

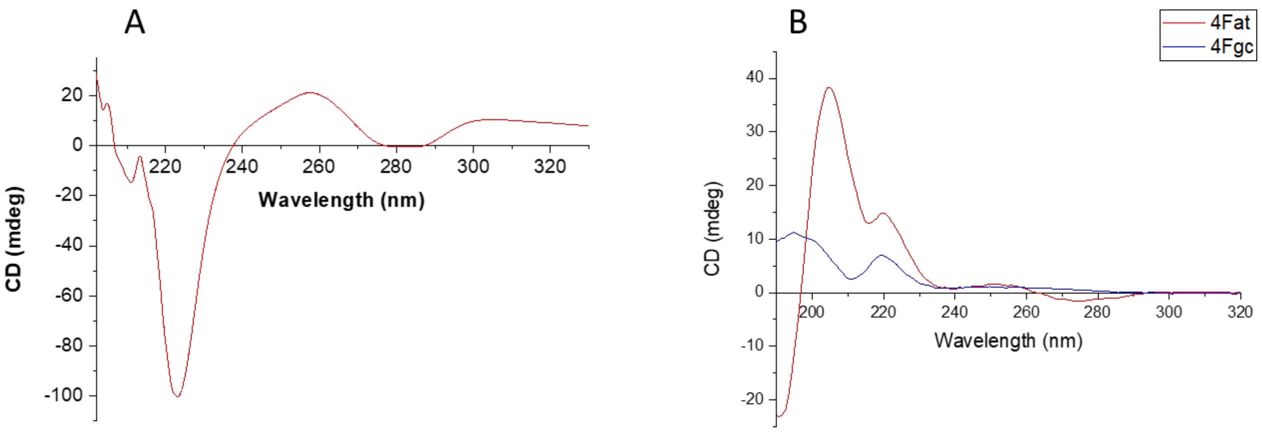

Figure S4: FT-IR spectra of 4Fat (top) and 4Fgc (bottom)

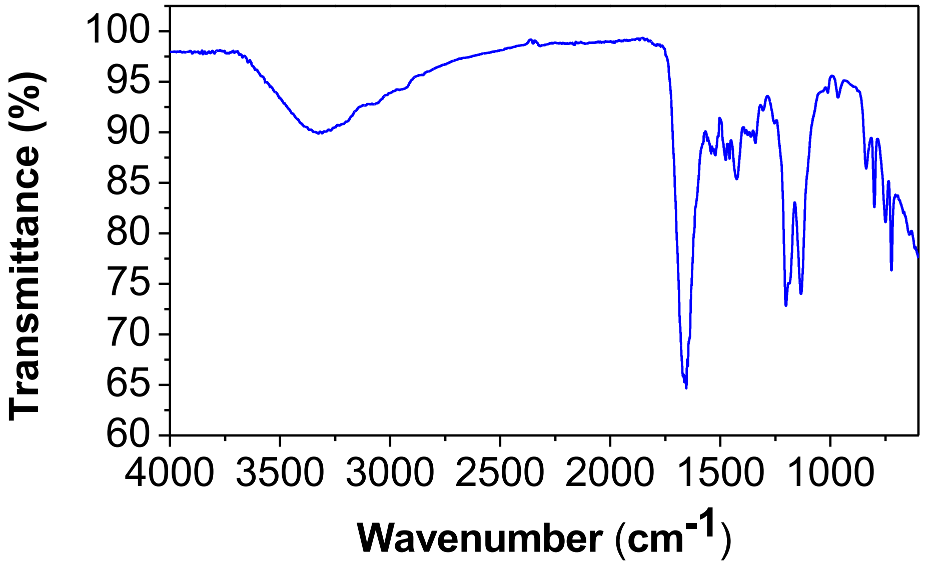

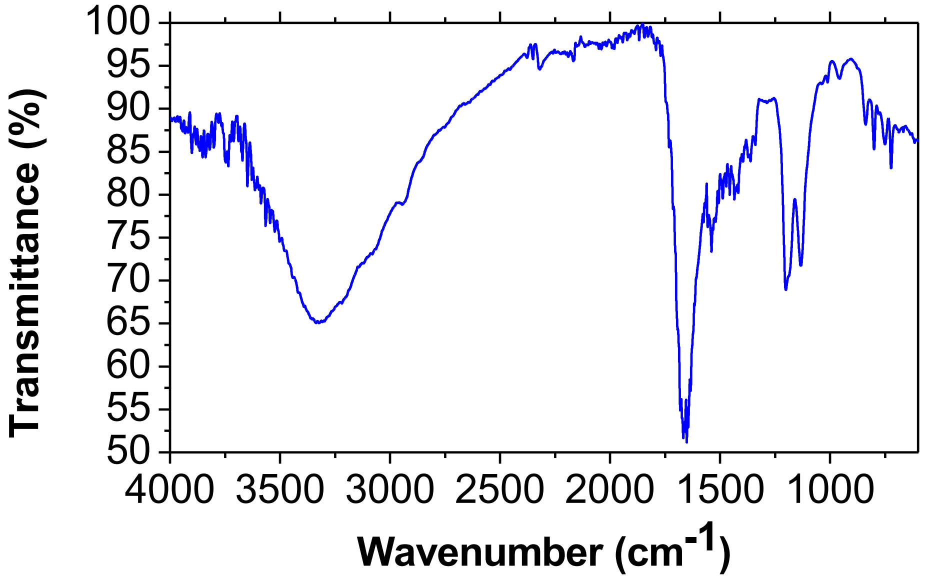

Figure S5: UV spectra of Congo Red (red), Congo Red+4Fat (blue), Congo red+4Fgc (green). Spectra are obtained upon subtraction of the dye and normalization.

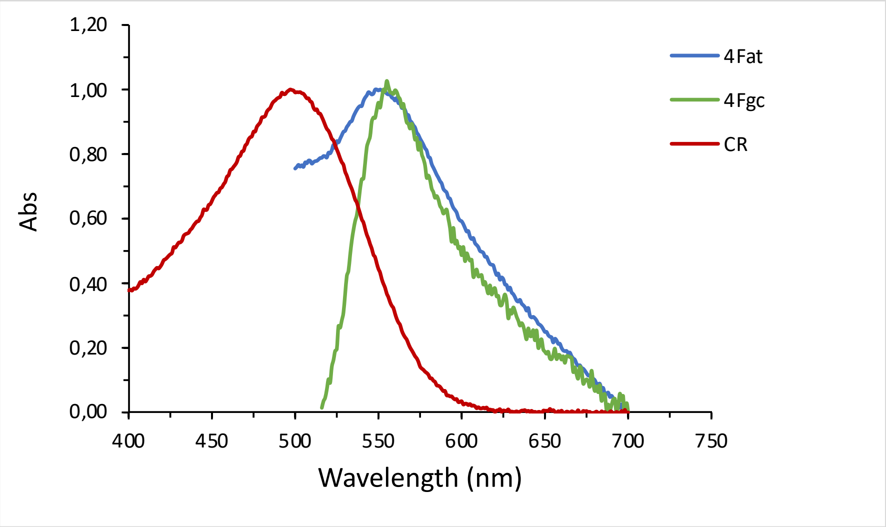

Figure S6: Fluorescence spectra: A and B: emission of 4Fat (A) and 4Fgc (B) upon excitation at 257 nm; C: excitation (black) and emission (red) spectra of 4Fgc (λ em= 430 nm, λ ex: 360 nm) in water 4Fgc D: emission spectra of 4Fgc upon excitation at 360 nm at different concentrations; E: solid state emission of 4Fgc upon excitation at 330-385 nm (top left), 488 nm (top right), 546 nm (bottom left), bright field (bottom right).

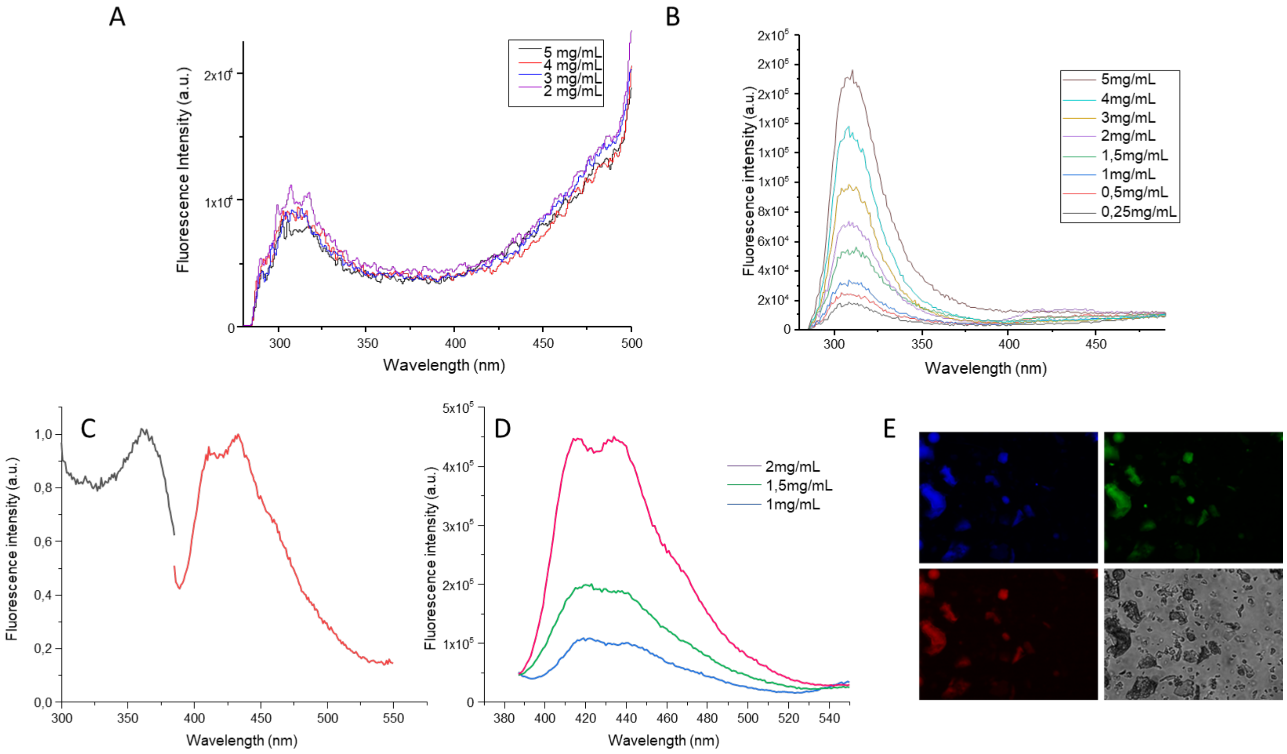

Figure S7: **CAC determination by fluorescence measurements**. For 4Fat fluorescence emission at 418 nm nm obtained upon excitation at 330 nm was plotted versus concentration (CAC= $1.6x10^{-5}$M). For 4Fgc fluorescence emission at 400 nm obtained upon excitation at 310 nm was plotted versus concentration (CAC=$3.4x10^{-5}$M).

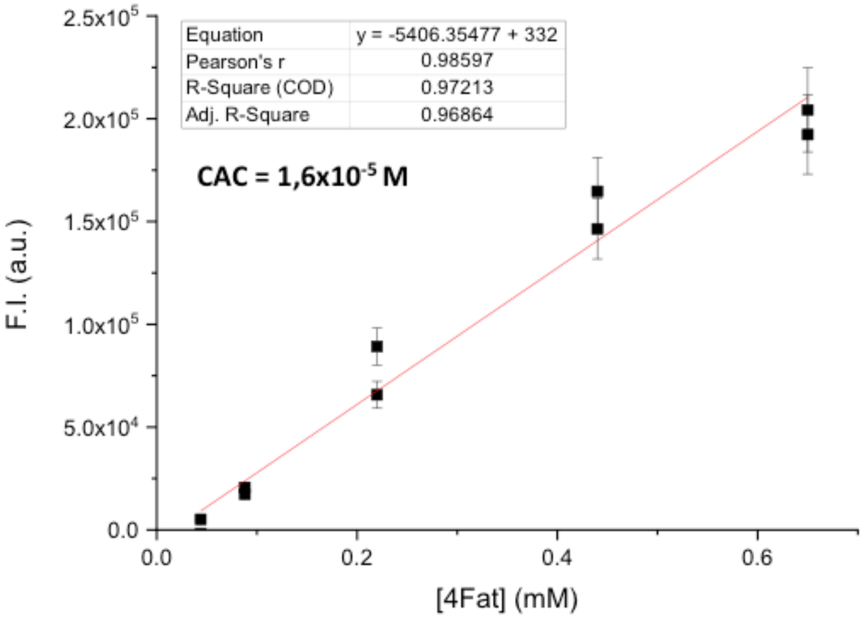

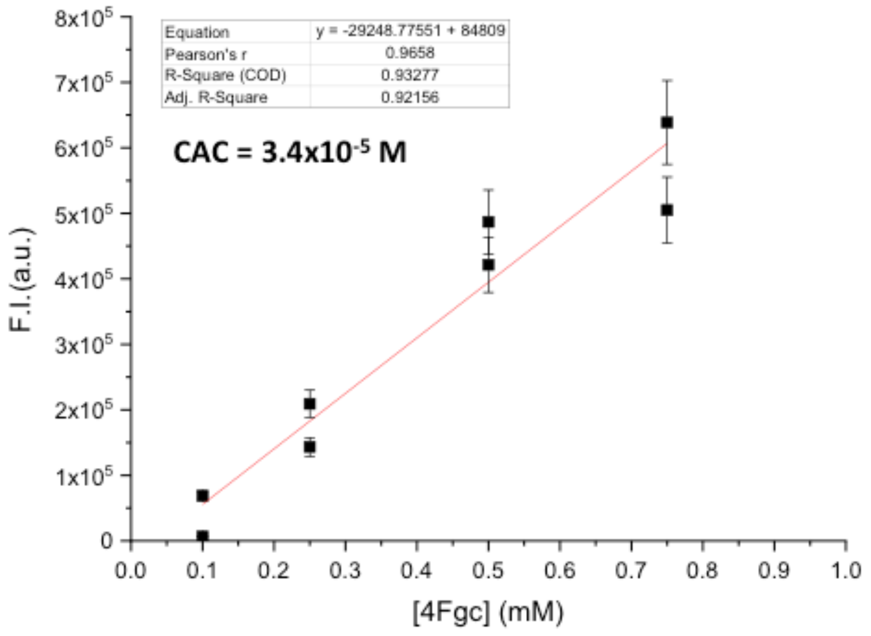

Figure S8: Tapping mode AFM images of the 4Fat (left) and analysis of the distribution of the 4Fat fibers' width (right).

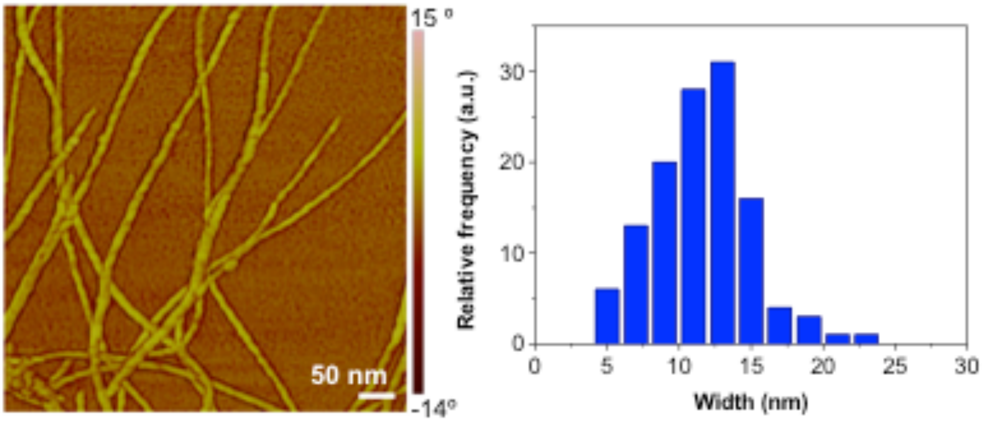

Scheme S1. Atom nomenclature.

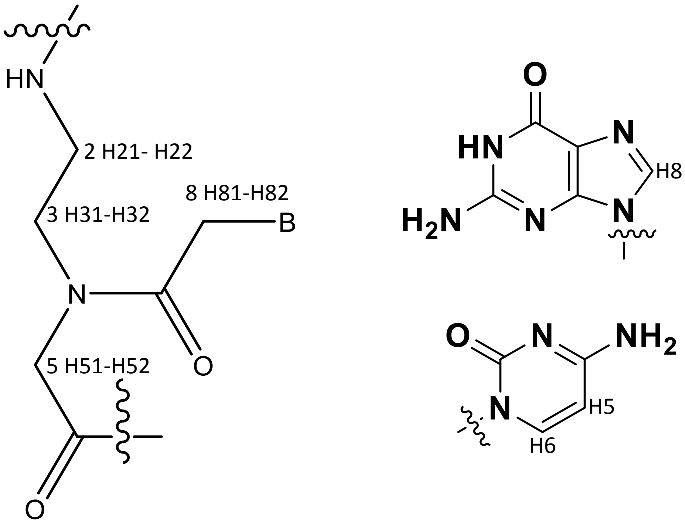

Scheme S2. Chemical structures of different PNA rotamers. The red box highlights the most abundant PNA rotamer.

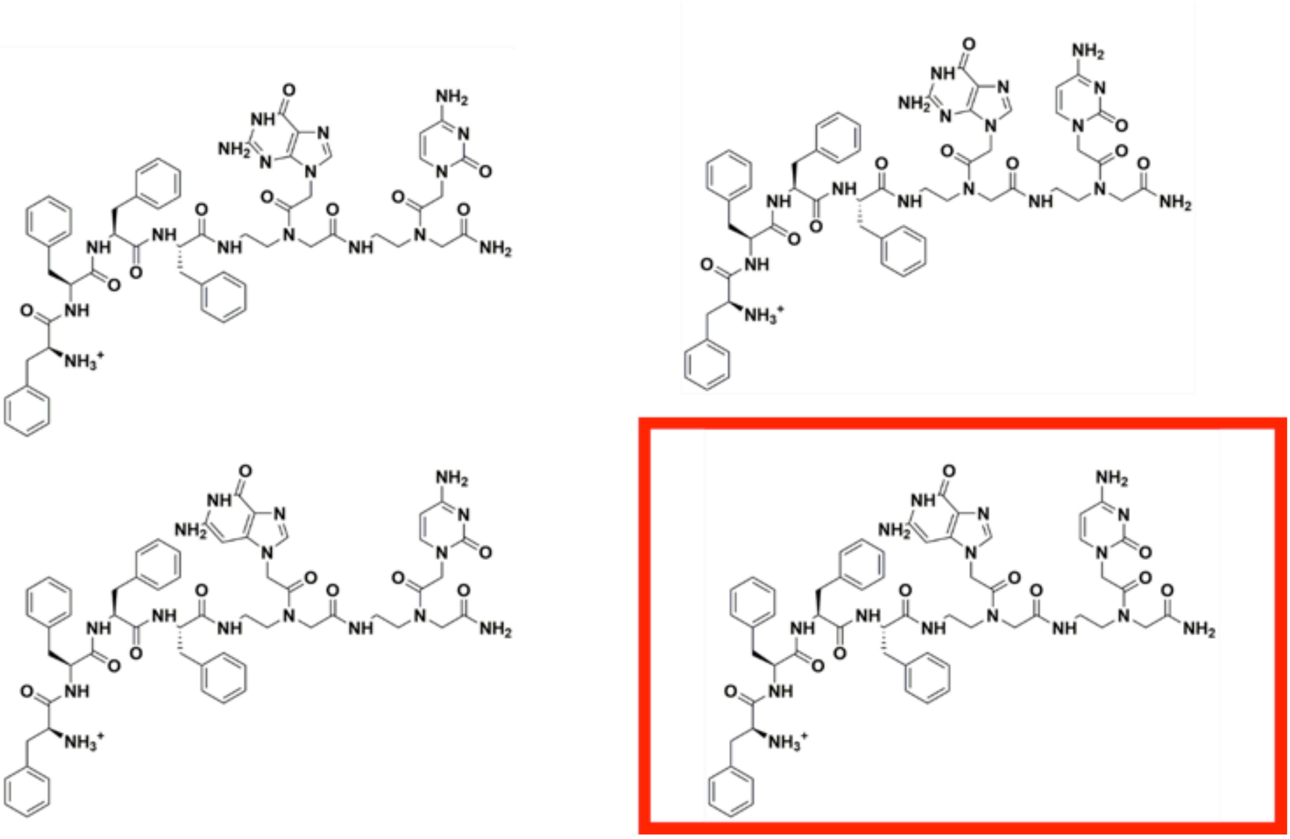

Figure S9. (Upper panel) Expansion of the 1D ^{1}H spectrum of 4Fgc recorded in H_2O/D_2O (90/10) showing signals of H5 aromatic protons from the cytosine four rotamers. (Lower panel) 2D [^{1}H-^{1}H] TOCSY spectrum of4Fgc: in the shown expansion cycles highlight correlations in between aromatic H5 and H6 cytosine protons in the four rotamers whereas, terminal $CONH_2$ groups are enclosed in the squares. The orange cycle and square highlight signals arising from the most abundant rotameric form.

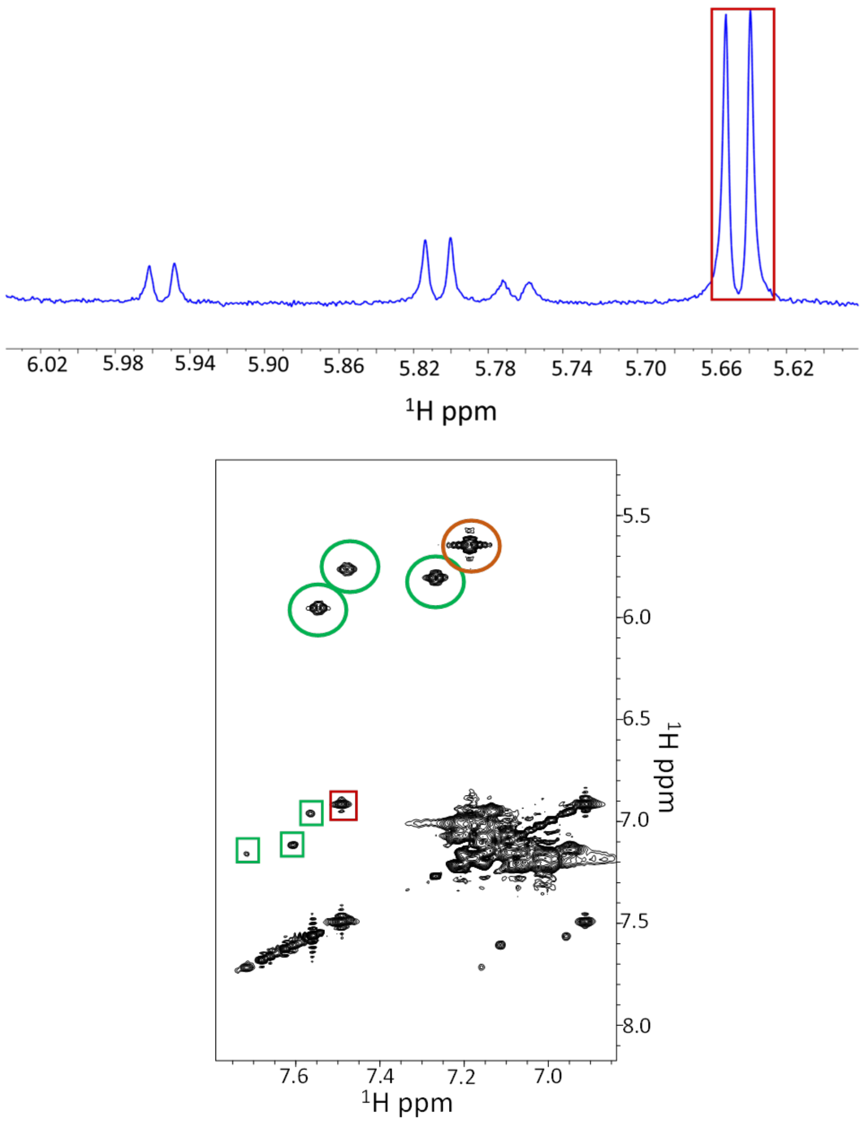

Figure S10. 2D [^{1}H, ^{1}H] NOESY300 spectrum of 4Fgc. In the shown expansion signals arising from aromatic protons of g5 and c6 are present along with contacts from phenylalanines aromatic protons (within the violet rectangle).

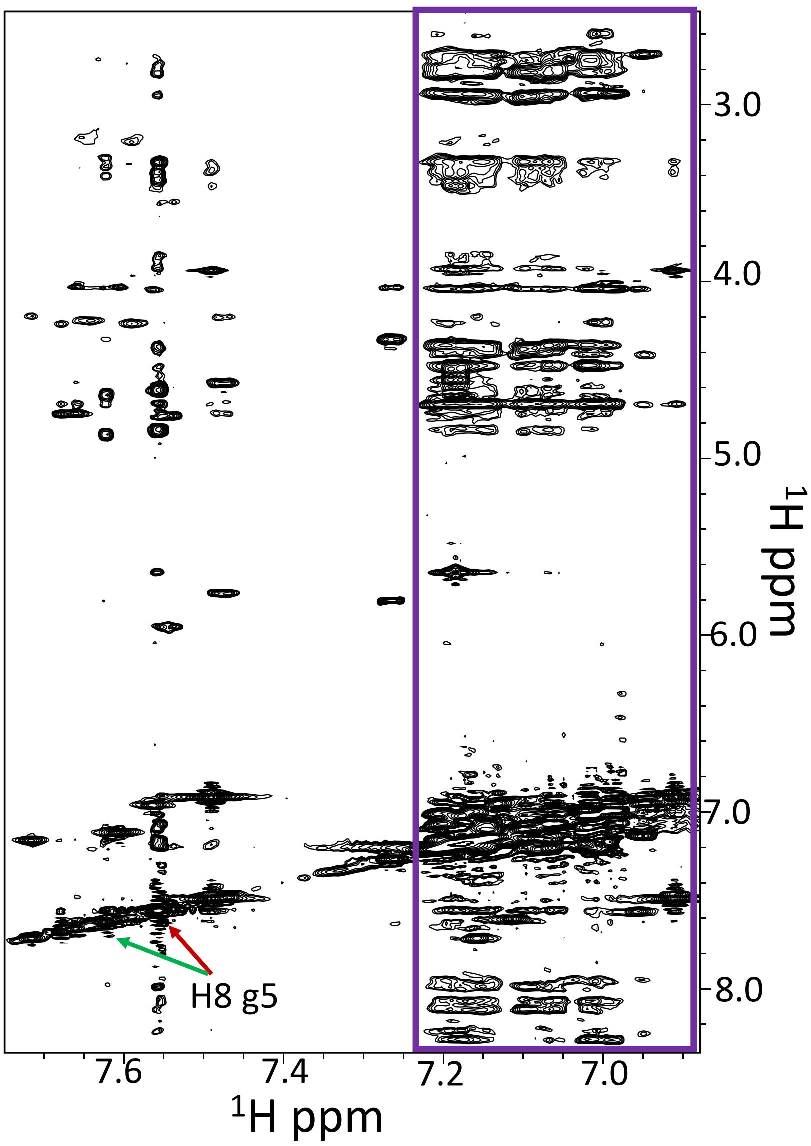

Figure S11. Comparison of backbone HN/high field correlation regions in TOCSY (left) and NOESY (right) spectra of 4Fgc. Assignments of spin systems are indicated for the major rotamer; the red arrows follow the main sequential Hαi-HNi+1 NOEs.

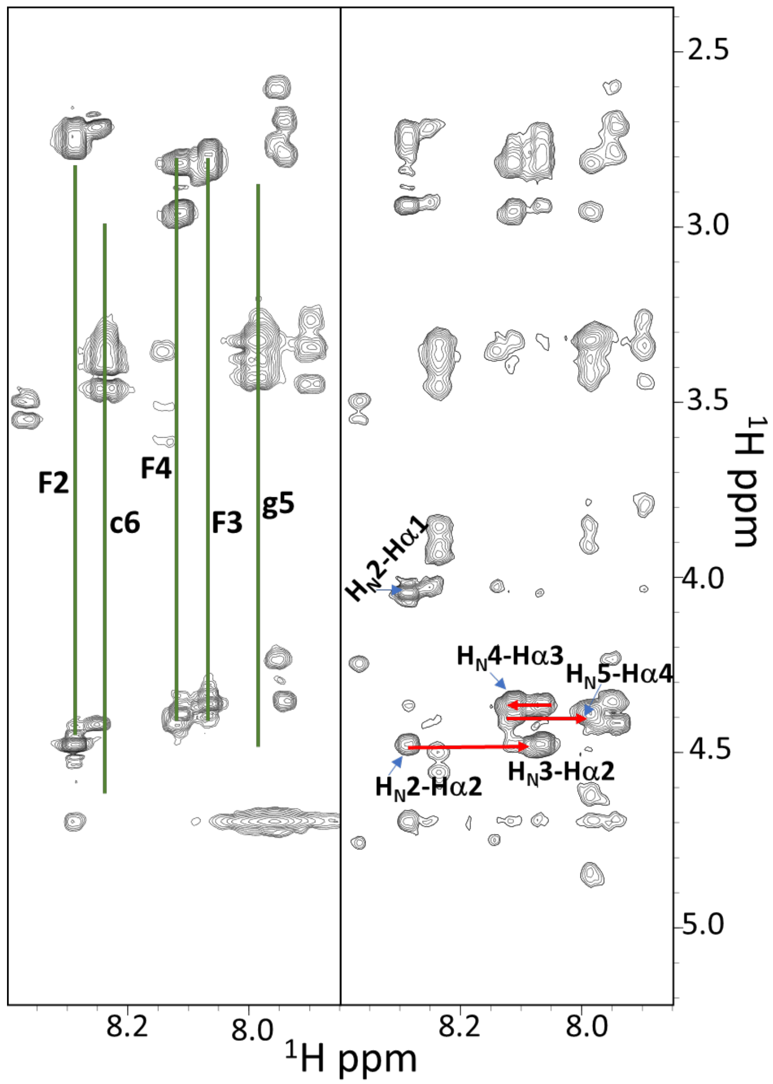

Figure S12. Region of the NOESY 300 spectrum showing principal correlations arising from H81-H82 aliphatic protons in g5 and c6. "Q5" indicates the chemical shift degeneracy between H5 CH2 protons in c6.

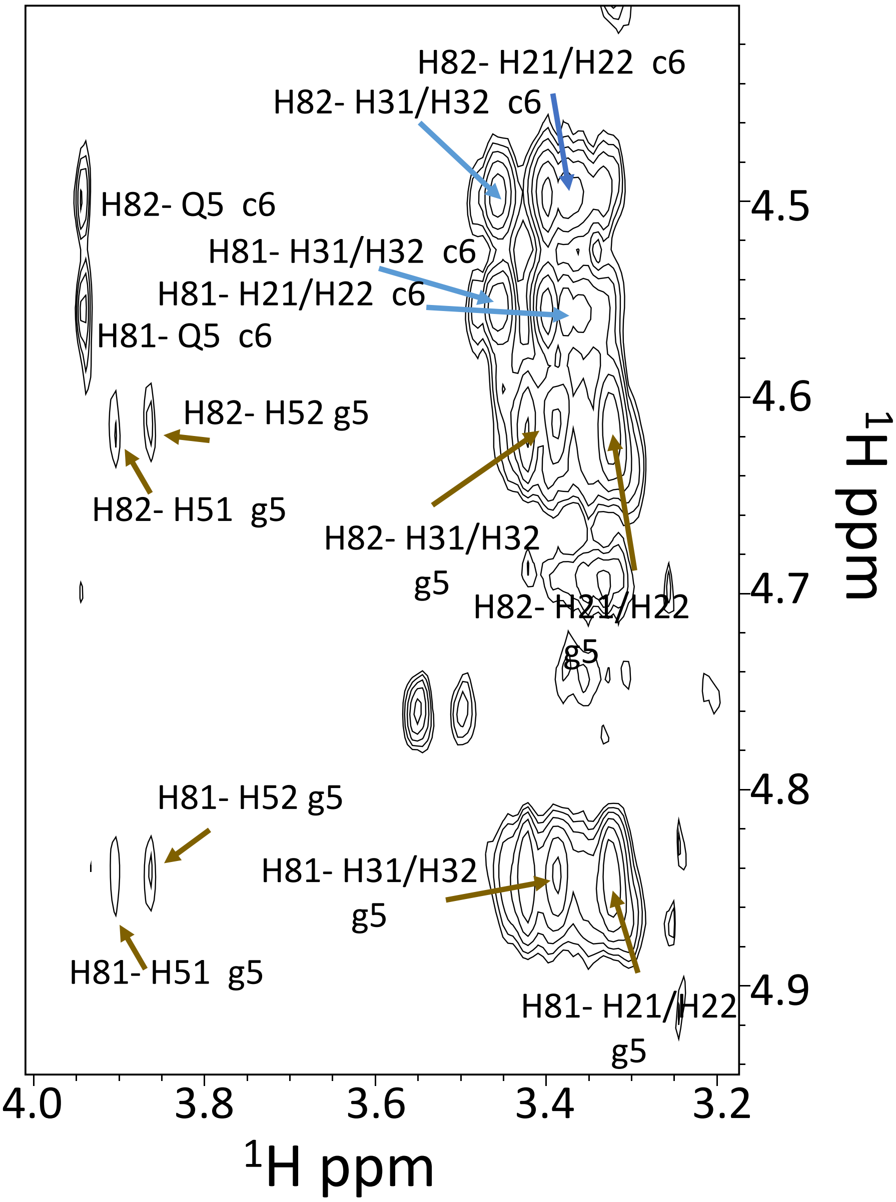

Figure S13A. Ensembles of different conformational families obtained through the ensemble cluster analysis of UCSF Chimera [1, 2]. Only the three most populated clusters are shown. Members of each cluster have been overlayed on all atoms in residues 1-6.

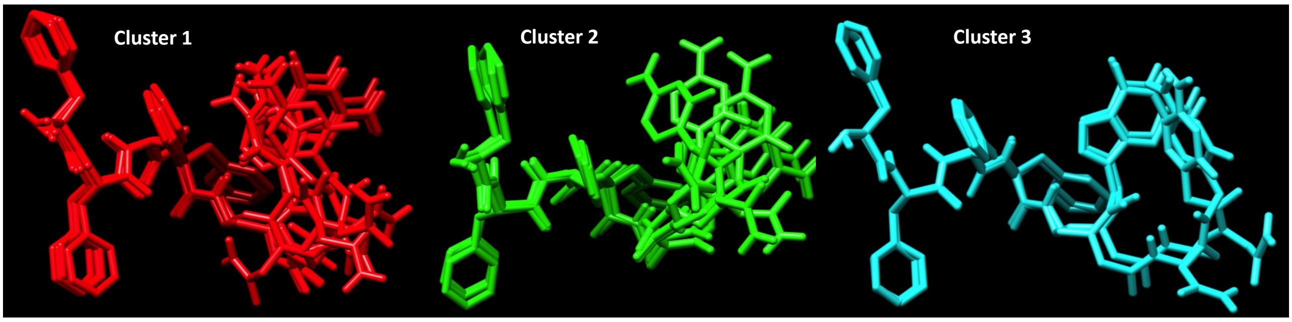

Figure S13B. CYANA structures n. 12, 1 and 16 are shown in the left, middle and right panels respectively. The shown conformers are the representative structures from the three most populated clusters [1, 2].

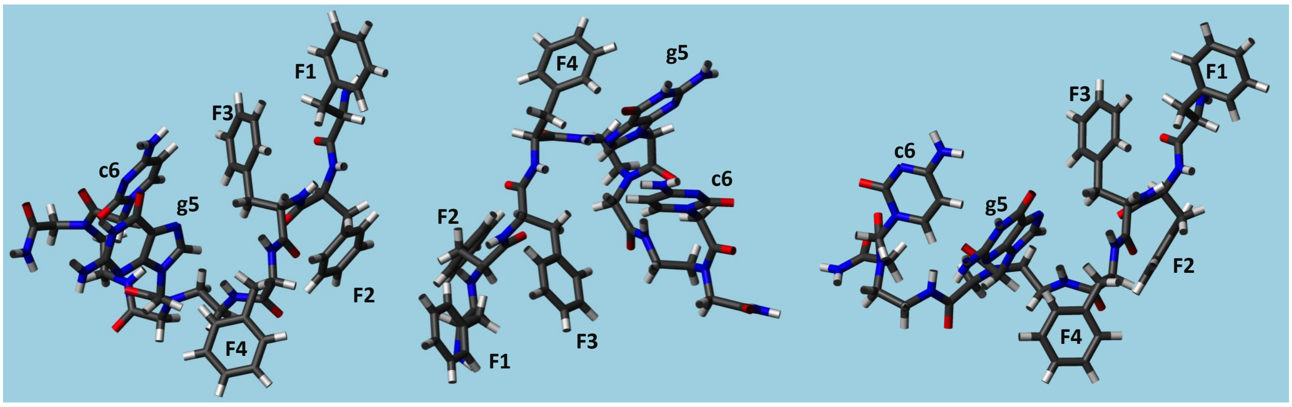

Figure S14. Representative model of the 4Fgc dimer obtained by molecular docking (docking score: -7.7 Kcal/mol) [39].

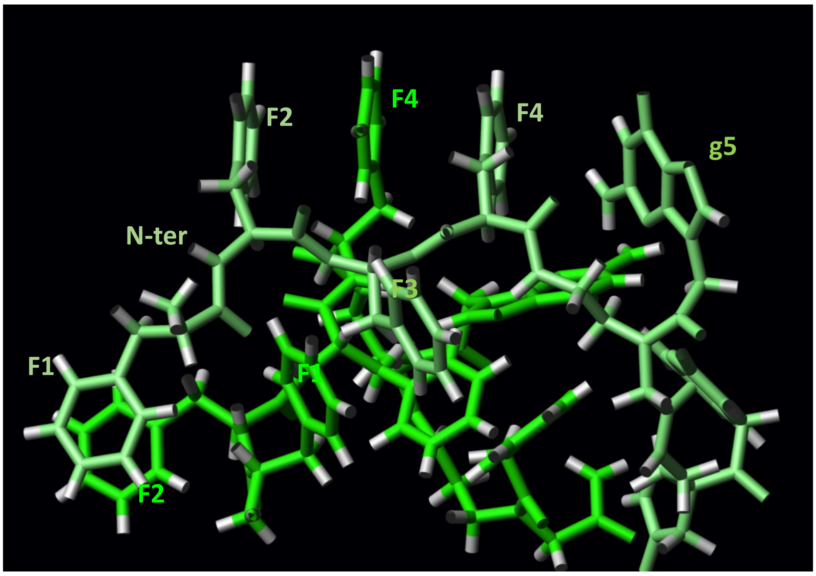

Table S1. ^{1}H Chemical shifts (ppm) of 4Fgc main conformer. Chemical shifts were referenced to water peak at 4.7 ppm. Ambiguous assignments due to spectral overlaps are reported in red.

Residue	H_N	Hα	Hβ			Others
**1 F**		4.04	2.949<br>2.932			Hδ1 6.99<br>Hδ2 6.99<br>Hε1 7.17<br>Hε2 7.17
**2 F**	8.285	4.473	2.778<br>2.719			Hδ1 7.02<br>Hδ2 7.02<br>Hε1 7.18<br>Hε2 7.18
**3 F**	8.063	4.36	2.827<br>2.794			Hδ1 7.07<br>Hδ2 7.06<br>Hε1 7.20<br>Hε2 7.20
**4 F**	8.113	4.385	2.965<br>2.832			Hδ1 7.10<br>Hδ2 7.09<br>Hε1 7.15<br>Hε2 7.15
	**H_N**	**H21-H22**	**H31-H32**	**H51-H52**	**H81-H82**	**Others**
**5 PG**	7.987	3.333-3.320	3.377-3.427	3.908-3.854	4.841-4.621	H8 7.558
**6 PC**	8.238	3.368-3.332	3.46-3.45	3.937	4.557-4.498	H5 5.647<br>H6 7.187<br>$CONH_2$ 7.491-6.907

Table S2. Cluster analysis of 4Fgc structures.

Cluster	Model numbers	Representative structure#
1	10-11-12-14-15	12
2	1-2-3	1
3	16-18	16
4	20	20
5	19	19
6	17	17
7	13	13
8	9	9
9	8	8
10	7	7
11	6	6
12	5	5
13	4	4

#Representative models retrieved by the clusterization procedure of Chimera [1] employing the approach described in Kelley et al. [2]

References

1. Pettersen, E. F.; Goddard, T. D.; Huang, C. C.; Couch, G. S.; Greenblatt, D. M.; Meng, E. C.; Ferrin, T. E. UCSF Chimera--a visualization system for exploratory research and analysis. *J Comput Chem* **2004,** *25*, 1605-12.
2. Kelley, L. A.; Gardner, S. P.; Sutcliffe, M. J. An automated approach for clustering an ensemble of NMR-derived protein structures into conformationally related subfamilies. *Protein Eng* **1996,** *9*, 1063-5.
